# Cerebrovascular Smooth Muscle Cells as the Drivers of Intramural Periarterial Drainage of the Brain

**DOI:** 10.3389/fnagi.2019.00001

**Published:** 2019-01-23

**Authors:** Roxana Aldea, Roy O. Weller, Donna M. Wilcock, Roxana O. Carare, Giles Richardson

**Affiliations:** ^1^Mathematical Sciences, University of Southampton, Southampton, United Kingdom; ^2^Clinical Neurosciences, Faculty of Medicine, University of Southampton, Southampton General Hospital, Southampton, United Kingdom; ^3^Department of Physiology, Sanders-Brown Center on Aging, University of Kentucky, Lexington, KY, United States

**Keywords:** lymphatic, brain, vasomotion, multi-scale model, poroelastic, Alzheimer's disease, cerebral amyloid angiopathy, perivascular drainage

## Abstract

The human brain is the organ with the highest metabolic activity but it lacks a traditional lymphatic system responsible for clearing waste products. We have demonstrated that the basement membranes of cerebral capillaries and arteries represent the lymphatic pathways of the brain along which intramural periarterial drainage (IPAD) of soluble metabolites occurs. Failure of IPAD could explain the vascular deposition of the amyloid-beta protein as cerebral amyloid angiopathy (CAA), which is a key pathological feature of Alzheimer's disease. The underlying mechanisms of IPAD, including its motive force, have not been clarified, delaying successful therapies for CAA. Although arterial pulsations from the heart were initially considered to be the motive force for IPAD, they are not strong enough for efficient IPAD. This study aims to unravel the driving force for IPAD, by shifting the perspective of a heart-driven clearance of soluble metabolites from the brain to an intrinsic mechanism of cerebral arteries (e.g., vasomotion-driven IPAD). We test the hypothesis that the cerebrovascular smooth muscle cells, whose cycles of contraction and relaxation generate vasomotion, are the drivers of IPAD. A novel multiscale model of arteries, in which we treat the basement membrane as a fluid-filled poroelastic medium deformed by the contractile cerebrovascular smooth muscle cells, is used to test the hypothesis. The vasomotion-induced intramural flow rates suggest that vasomotion-driven IPAD is the only mechanism postulated to date capable of explaining the available experimental observations. The cerebrovascular smooth muscle cells could represent valuable drug targets for prevention and early interventions in CAA.

## 1. Introduction

The brain lacks a conventional lymphatic system, which in the rest of the body is responsible for removing waste products and excess fluid (Aspelund et al., 2015; Louveau et al., 2015; Bakker et al., 2016). Of high interest is the clearance of soluble amyloid-beta (Aβ) proteins that are released by neurons into the surrounding extracellular spaces (i.e., interstitium) following normal synaptic activity (Tarasoff-Conway et al., 2015). Inefficient removal of Aβ from the brain leads to parenchymal amyloid plaques and cerebral amyloid angiopathy (CAA), commonly seen in the commonest form of dementia, Alzheimer's disease (AD) (Selkoe, 2001; Ross and Poirier, 2005; Cupino and Zabel, 2014). CAA describes the accumulation of Aβ initially within the basement membranes (BMs) of vascular smooth muscle cells (VSMCs) of cortical and leptomeningeal arteries, but also within the BM of cortical capillaries (with or without recruitment of other vessels). Although CAA has been almost invariably reported in AD, the exact causes of the onset of CAA remain unclear (Charidimou et al., 2012). The hemorrhages and ischemic lesions associated with CAA result in cognitive impairment and dementia (Attems et al., 2011; Reijmer et al., 2015). Currently, 47 million people worldwide suffer from dementia and there is no effective curative or preventive intervention. Aging highly increases the risk for CAA and dementia and, considering increased longevity, the occurrence of dementia by 2050 is evaluated to 131 million people (Prince et al., 2016). This increases the urgency to explore potential unconventional clearance mechanisms for Aβ that may contribute toward maintaining the homeostasis of the brain (Tarasoff-Conway et al., 2015; Bakker et al., 2016).

Aβ produced by neurons is degraded by enzymes (Farris et al., 2003; Marr et al., 2003), transported into the blood via lipoprotein receptor related protein (LRP)-1 (Deane et al., 2008) or cleared along the walls of capillaries and arteries (Hawkes et al., 2014). The Aβ transport along the wall of arteries, generally termed perivascular drainage, has been a subject of considerable controversy over the last few years. The pathways for the elimination of interstitial fluid (ISF) and solutes (including Aβ) from the brain parenchyma have recently been reviewed by Abbott et al. (2018) and Hladky and Barrand (2018). Early experiments by Szentistvanyi et al. (1984) showed that the major pathway by which radioactive solutes drain from the brain to cervical lymph nodes is along the walls of cerebral arteries in the direction counter to the blood flow. This pathway was later located in the BMs interposed between the VSMCs from the tunica media of cerebral arteries (Carare et al., 2008), i.e., the Intramural Peri-Arterial Drainage (IPAD) pathways, effectively the lymphatic drainage routes of the brain (Carare et al., 2014; Morris et al., 2014). An alternative viewpoint has also been postulated which states that, following influx of cerebrospinal fluid (CSF) and solutes from the subarachnoid space into the brain parenchyma, the CSF-ISF mixture is cleared along the walls of intracortical veins, back into CSF (Iliff et al., 2012). However, apart from the original paper, there is little support for this para-venous clearance mechanism. Other studies that injected soluble tracers intracerebrally (Carare et al., 2008; Arbel-Ornath et al., 2013) or into the CSF (Albargothy et al., 2018) have found no evidence of tracer presence along the intracerebral para-venous pathways, but rather along the walls of cerebral arteries, specifically within the IPAD pathways.

**Figure 1:**
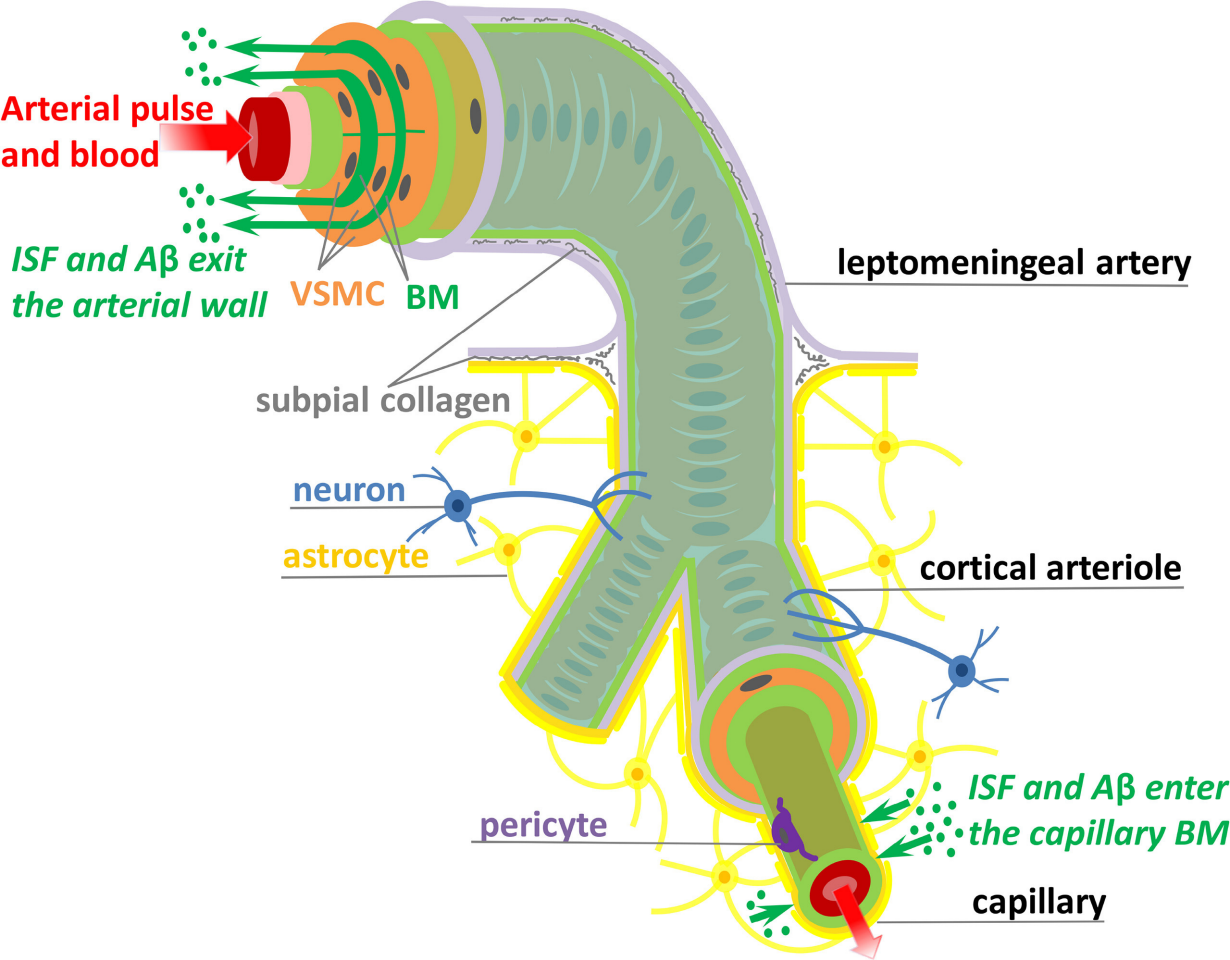
Schematic representation (not to scale) of the IPAD pathways. Soluble Aβ (green dots) and ISF from the brain interstitium enter the BM of capillaries and flow upstream toward large arteries through the BM (dark green) positioned between VSMCs (orange). Transport along the IPAD pathways is shown by the green arrows which are against the direction of arterial pulse and blood flow (red arrow). Three concentric layers of VSMCs are pictured at the larger extreme of the artery, while, for the sake of simplicity, only the outer most layer of VSMCs is shown along the length of the artery. The artery is wrapped in a pial sheath (light purple) and innervated by neurons (blue). At the capillary level, the endothelial BM (light green) is fused with the BM of glia limitans (dark yellow) secreted by astrocytes (yellow); they also interact with brain interstitium (due to gap junctions between the end-feet of astrocytes), allowing entrance of ISF and Aβ in the vascular wall (Morris et al., 2016; Weller et al., 2018). VSMC, vascular smooth muscle cell; BM, basement membrane; endothelium (red), internal elastic lamina (pink), inner and outer BM (light green), pericyte (purple), and subpial collagen (gray).

Here, we are concerned with shedding light on the mechanisms responsible for transport of ISF and Aβ along the IPAD pathways. The IPAD pathways were mapped by Carare et al. (2008) based on the following observations: soluble tracers injected into the brain parenchyma (i) rapidly enter the BM of capillaries and (ii) are progressively observed in BM in the tunica media of intracerebral arterioles and arteries, and later on, in the walls of leptomeningeal arteries (see Figure 1). Extensive work has been done for assessing the transport of solutes out of the brain along the IPAD pathways during aging (Hawkes et al., 2011) and under various physiological and pathological conditions, such as the presence of CAA (Arbel-Ornath et al., 2013; Hawkes et al., 2014), possession of APOE4 (Zekonyte et al., 2016), consumption of a high-fat diet (Hawkes et al., 2015) and after ischemic stroke (Arbel-Ornath et al., 2013). All those experiments showed that soluble tracers injected into the brain interstitium reach the BM of intracerebral and leptomeningeal arteries and their distribution within the arterial wall resembles the pathological deposition of Aβ in CAA (Weller et al., 1998; Preston et al., 2003). These findings indicate that soluble Aβ can be removed from the brain tissue along the IPAD pathways, in the opposite direction to arterial pulsations, and that failure of this clearance mechanism results in the vascular deposition of Aβ as CAA.

Improvement of IPAD holds great promise for treatment of CAA. However, significant advancement is unlikely until the underlying mechanisms of IPAD, including the motive force, are elucidated. The observation that no periarterial drainage occurs following cardiac arrest suggested that arterial pulsations, derived from the heartbeat, might drive IPAD in the brain (Carare et al., 2008). Driving net intramural periarterial flow in the opposite direction to the arterial pulse, as shown in Figure 1, appears to be physically difficult. Several mechanisms that could allow periarterial clearance to occur in the reverse direction to arterial pulsations have been mathematically modeled, all of them assuming the arterial pulse to be the motive force for IPAD (Schley et al., 2006; Coloma et al., 2016; Sharp et al., 2016; Diem et al., 2017). Previous suggestions included some degree of attachment between solutes and BM (Schley et al., 2006), different flexible structures within the BM (Sharp et al., 2016) and a valve mechanism (Diem et al., 2017), that would be needed to generate greater resistance to forward periarterial flow than to retrograde periarterial flow (against the direction of arterial pulse). The presence of flexible and valve-like structures within the wall of cerebral arteries still awaits experimental confirmation. More importantly, Diem et al. (2017) have shown that arterial pulsations are incapable of driving intramural fluid flow rates out of the brain of physiological significance, even with valve-like structures within the BM. The very long wavelength of the arterial pulse is inadequate to induce pressure gradients large enough to generate periarterial flow rates comparable with experimental observations (Carare et al., 2008; Arbel-Ornath et al., 2013). Forces other than cardiac pulsations must therefore drive IPAD in the brain and the quest for other candidates is still open.

With this in mind, we propose that the forces generated by cerebral VSMCs can drive IPAD in the brain by acting upon the deformable BM. The VSMCs are contractile cells embedded within the arterial wall and, under physiological conditions, generate a basal vascular tone that is maintained by a combination of various stimuli (e.g., arterial pressure, shear stress, neuronal metabolic activity and several types of innervation) (Cipolla, 2009). Deviations from the basal vascular tone result in significant variations in the diameter of arteries. The evoked vasomotor response is able to spread along the artery length, as well as across branch points (Welsh et al., 2018). The conducted vasomotor response may be induced by vasoactive drugs or it may occur spontaneously. The spontaneous rhythmic oscillations of vascular tone are known as vasomotion (Nilsson and Aalkjær, 2003; Aalkjær et al., 2011). The initial, local contractions of the VSMCs are propagated over macroscopic distances as a contraction wave that is linked to the calcium waves mediated via intercellular gap-junctions (Seppey et al., 2009; Pradhan and Chakravarthy, 2011). Vasomotion is independent of pulse rate and respiration and has been observed in the vascular beds of numerous tissues, including the cerebral tissue (Fujii et al., 1990, 1991; Gokina et al., 1996; Mayhew et al., 1996; Obrig et al., 2000; Filosa et al., 2004; Vetri et al., 2007). Vasomotion has been previously modeled as a mechanism aiding the transport of blood (Carew and Pedley, 1997; Ursino et al., 1998) and oxygen (Goldman and Popel, 2001; Hapuarachchi et al., 2010) from blood vessels to various tissues in the body. Di Marco et al. (2015) reviewed the ways in which cerebral vasomotion may be hindered in AD and mentioned that vasomotion could act as a secondary input to the motive force of arterial pulse-driven IPAD. A systematic contribution of the cerebral VSMCs to the periarterial drainage of fluid out of the brain has not been previously inspected, neither experimentally nor by any modeling technique.

**Figure 2:**
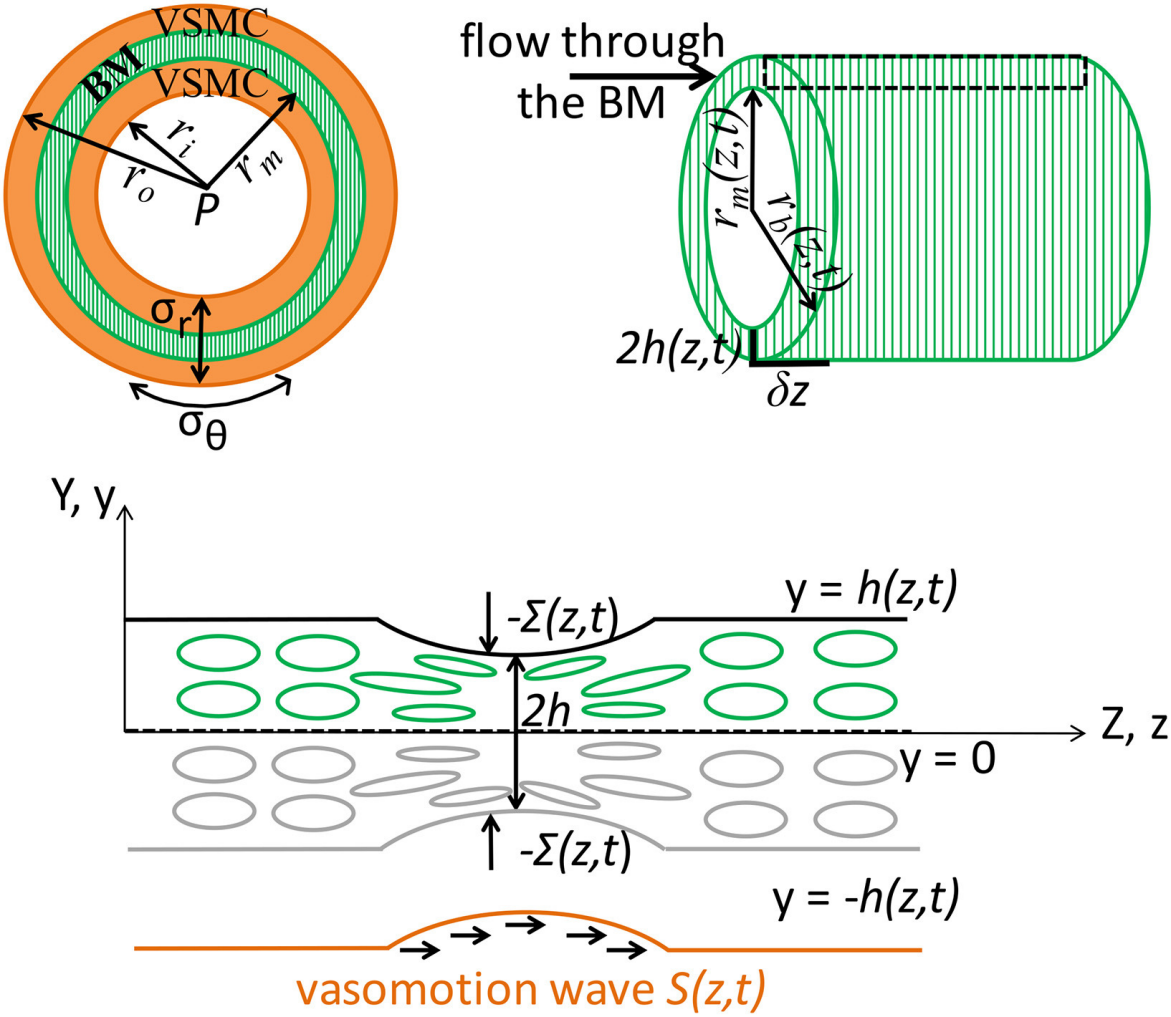
Schematic representation (not to scale) of the V-IPAD model. A leptomeningeal artery is modeled as a long thick-walled cylinder with uniform material properties along its length, maintained at a constant intraluminal pressure *P* and constant longitudinal stretch and exposed to active contractions of VSMCs. The top layer shows the arterial cross section with a layer of BM (green compartment) embedded in the wall **(Left)** and the longitudinal section of the BM **(Right)**. For simplicity reasons, only one layer of BM is considered at the middle of the wall and the two layers of the VSMCs surrounding the BM are assumed to behave identically. The remaining wall components are not shown, but their effect on the wall elasticity is captured by the radial (σ_*r*_) and circumferential stress (σ_θ_). The deformed inner radius, middle radius and outer radius of the arterial wall are denoted by *r*_*i*_, *r*_*m*_, and *r*_*o*_, respectively. The position of the inner layer bounding the BM is assumed at *r*_*m*_ and, owing to stress continuity across the wall, the radial stress σ_*r*_ at that point represents the external compressive stress Σ which acts on the BM, i.e., Σ = σ_*r*_(*r* = *r*_*m*_); thereby, the arterial wall model is coupled with the BM model. Both *r*_*m*_ and Σ depend on the prescribed vasomotion wave *S*(*z, t*) generated by the contractile VSMCs and are determined from the arterial wall model. The position of the outer layer bounding the BM is assumed at *r*_*b*_ with *r*_*b*_ = *r*_*m*_ + 2*h*, where 2*h* denotes the whole BM thickness. Since the BM thickness is significantly smaller than the arterial radius, its upper half is assumed to behave identically to its lower half. The deformed thickness *h* = *h*(*z, t*) of the upper-half BM is determined by solving the BM model in the Cartesian system (*y, z*) and this is justified by the relationship *r*_*b*_ = *r*_*m*_(*z, t*) + 2*y* where 2*y* ≪ *r*_*m*_ and 2*h* ≪ *r*_*m*_. The area delimited by the rectangular is illustrated in the bottom schematic: a sheet of BM modeled as a poroelastic compartment whose fluid-filled pores are squeezed during the VSMC contractions, under the direct actions of −Σ(*S*(*z, t*)), driving the fluid out of the BM pores in the direction of the vasomotion wave.

Proposed mechanism: vasomotion-driven IPAD. We propose the hypothesis of vasomotion-driven IPAD (denoted V-IPAD) and explain below the underlying mechanisms for this potential clearance process. The position of the contractile VSMCs within the artery wall appears to be ideal for allowing an immediate effect of their contractions on the conformation of the adjoining BM, which is a specialized sheet of extracellular matrix filled with interstitial fluid (see Figure 2). In other words, the BM is a soft fluid-filled poroelastic medium whose pores could be closed and reopened during VSMC contraction and relaxation, respectively. In this way, the contractions of two layers of VSMCs will squeeze the interposed BM, pushing fluid out of its pores in the direction of the contraction wave. In order to clear any soluble material from the brain interstitium via the IPAD pathways, the vasomotion wave must propagate from the intracerebral arterioles toward the large arteries on the surface of the brain. The V-IPAD mechanism bears some analogy to an old-fashioned mangle or a wringer which squeezes water out of soft materials such as towels. Other parts of the human body, such as the digestive system, use wave-like muscular contractions to propel the content of a tube (e.g., food along the gastro-intestinal tract); this process is also known as peristaltic pumping. We note that the nomenclature of “peristalsis” has also been used in modeling studies of perivascular drainage driven by the heart-derived arterial pulse (Bilston et al., 2003; Wang and Olbricht, 2011; Sharp et al., 2016), rather than by the VSMCs. However, the vasomotion wave has significantly different properties than the arterial pulse. For example, vasomotion has a wavelength of several millimeters, which is at least two orders of magnitude lower than that of arterial pulsations. In addition, the arterial pulse has an approximate frequency of 1 Hz, while the vasomotion frequency, although varies with tissue type and species, is commonly taken to be 0.1 Hz (Nilsson and Aalkjær, 2003; Aalkjær et al., 2011). Hence, comparison between the current work and previous studies should be made with caution because both the arterial pulse and the vasomotion wave are commonly modeled as sinusoidal waves.

Here, we test the role of cerebral vasomotion in the clearance of fluid from the brain by developing a novel physiologically-based multiscale model of a middle cerebral artery (MCA); this model is denoted the V-IPAD model and presented in section 2.1 (see details in Aldea, 2017). The V-IPAD model couples two models: (i) the arterial wall model which captures the biomechanics of an active elastic cerebral artery and (ii) the BM model which yields the fluid flow rates, along the IPAD pathways, generated by the activation of the VSMCs. We emphasize that this is the first model treating the BM as a fluid-filled poroelastic medium, rather than just a fluid-filled channel (Schley et al., 2006; Coloma et al., 2016) or a fluid-filled porous medium (Wang and Olbricht, 2011; Diem et al., 2017).

The poroelastic BM is a biphasic material composed of: (i) a porous and elastic solid matrix (e.g., the extracellular matrix of BM proteins) and (ii) a fluid component (e.g., the ISF) that occupies the connected pores, an example being a water filled bath sponge. Where the solid matrix deforms in response to an external load it transmits force (in the form of a pressure) to the fluid filling the pores; this subsequently leads to changes in the permeability of the solid matrix to fluid flow as, for example, the pores close up. As it will be shown, the elastic deformations of the poroelastic BM, induced by the contractile VSMCs, are critical in assuring high net fluid flow rates along the IPAD pathways, without the need of any intramural valves; such a behavior could not be obtained in a purely porous, underformable material. Models of poroelasticity have been widely applied to biological materials in applications ranging from fluid movement in bone (Cowin, 1999), to tumor growth (Roose et al., 2007) and biomechanics of brain tissue (Goriely et al., 2015).

The mathematical details of the V-IPAD model are given in section 2. The simulation results are presented in section 3, followed by their discussion in section 4. We have written the paper such that the sections 3 and 4 can be read prior to section 2. In section 4, we also discuss suitable animal models (e.g., hyperhomocystaeinaemia (HHcy) mouse models) for testing experimentally the V-IPAD hypothesis. In Supplementary Material 1, we present in detail the arterial wall model which follows popular hyperelastic arterial models from the literature (Rachev and Hayashi, 1999; Kalita and Schaefer, 2008), while in Supplementary Material 2, we explain the numerical implementation of the BM-model. Finally, our pilot experimental data from the HHcy animal models are shown in Supplementary Material 3.

## 2. Materials and Methods

In this section, we first describe the governing equations of the V-IPAD model and then explain their physiological significance. The V-IPAD model consists of two coupled models: (i) the BM model for the vasomotion-induced intramural periarterial flows through the deformable poroelastic BM, coupled to (ii) the arterial wall model for the elastic response of a rat MCA. We recall that the latter model is presented in detail in Supplementary Material 1, so below we focus on the novel BM model. We also present a concise formulation of the BM model that is solved numerically in Supplementary Material 2. The full derivation of the BM model can be found in Aldea (2017). The parameters used in the V-IPAD model are given in Table 1.

**Table 1:** Dimensional parameters.

**Parameter**	**Value**	**Unit**	**Description**	**References**
*H*	0.20	μm	Thickness of upper-half BM	
*L*_*a*_	2,000	μm	Artery length	Bell et al., 2013
*L*_*s*_	10	mm	Computational system length	
$\phi_{*}^{s}$	0.25	1	Solid volume fraction	Candiello et al., 2010
$\phi_{*}^{f}$	0.75	1	Fluid volume fraction	Candiello et al., 2010
μ_*s*_	3,700	Pa	Lamé parameter “lymphatic”	Heppell et al., 2013
λ_*s*_	8,600	Pa	Lamé parameter “lymphatic”	Heppell et al., 2013
k_*_	10^−2^	μm^2^	BM permeability	Heppell et al., 2013
κ	4/3	1	Parameter	Wirth et al., 2010
η	10^−3^	*Pa*·*s*	ISF viscosity	Syková and Nicholson, 2008
*S*_*m*_	10^5^	*Pa*	Maximum vascular tone	Rachev and Hayashi, 1999
*A*	1	1	Amplitude activation wave	
λ_*w*_	2,000	μm	Vasomotion wavelength	*c*_*w*_·*T*
*T*	10	*s*	Vasomotion period	Aalkjær et al., 2011
*c*_*w*_	200	μm · s^−1^	Average wave speed	Duling and Berne, 1970
				Dietrich et al., 1996; Seppey et al., 2009

### 2.1. Lubrication Model of the Poroelastic BM

The aim of this model is to quantify the amount of fluid eliminated from the brain tissue along the intramural vascular BM as a consequence of muscular contractions of cerebral arteries. The intramural vascular BM is modeled as a slowly varying sheet of width 2*h*, running through the wall of a cylindrical vessel which is itself centered along the z-axis, undergoing axisymmetric deformation (i.e., no θ-dependence), as illustrated in Figure 2. On its top and bottom boundaries (both assumed impermeable), the BM is exposed to compressive stresses dependent on the contractile activity of the VSMCs. Given its anatomical properties (e.g., a thin, extracellular matrix of fibrous proteins), the BM is treated as deformable spongy material filled with interstitial fluid. More specifically, the BM is modeled as a fluid-filled poroelastic medium comprised of a porous solid phase (the matrix of proteins) denoted by the superscript “s” and a fluid phase (interstitial fluid) denoted by the superscript “f.” The pores in the solid matrix provide a path for the movement of fluid. Since the BM thickness (≈ 0.4 μm) is significantly smaller than the arterial radius (≈ 100 μm), we assume that its upper half behaves identically to its lower half. For visual purposes, the following notation is adopted: 2*H* is the undeformed thickness of the BM and 2*h* is the deformed thickness of the BM.

The poroelastic BM is a compressible elastic medium subjected to deformations in response to an external compressive stress and to changes in fluid pressure in the pores of the matrix. Specifically, the external compressive stress, denoted Σ, is a known input function of time and position, i.e., Σ = Σ(*z, t*); it depends on the contractile oscillations of the VSMCs and its value is previously determined from the elastic analysis of an active cerebral artery, rather than just being a prescribed function from the literature (see Supplementary Material 1 and Aldea, 2017). Σ(*z, t*) affects the fluid flow through the BM by inducing deformations of its boundaries. Thus, the system depends on time only through the boundary conditions. Accounting for the symmetry of the system, as illustrated in Figure 2, it suffices to solve the BM model in the upper half plane in order to determine the deformed BM thickness (Aldea, 2017).

#### 2.1.1. Governing Equations

In general, solving a three-dimensional poroelastic model results in a highly complex system of equations. However, by exploiting the disparity between the length scales of the contracting arterial wall [BM thickness (0.4 μm) ≪ arterial radius (100 μm) ≪ wavelength of muscular contractions (2000 μm)], we derive a simplified version of the BM model by using a lubrication approximation (see details in Aldea, 2017). Thereby, it is conceivable to assume that the variations in the radial direction of the BM are weak and the variables of the system, at leading order, have only one spatial dependence, e.g., the axial z-dependence. This also makes it sensible to assess the BM model in a two-dimensional Cartesian system (*y, z*), where the Cartesian y-coordinate is related to the cylindrical r-coordinated by the relationship *r*_*b*_ = *r*_*m*_(*z, t*) + 2*y*, where 2*y* ≪ *r*_*m*_ and 2*h* ≪ *r*_*m*_; here, the expressions *r* = *r*_*b*_(*z, t*) and *r* = *r*_*m*_(*z, t*) describe the radial position of the outer and inner layer bounding the BM, respectively, as illustrated in Figure 2.

In order to quantitatively determine the fluid flow rate *Q*_*BM*_(*z, t*) through the BM, as an effect of VSMC activity, we first need to solve the system described below in Equations (1–5) for the following five variables: the deformed thickness of the BM *h*(*z, t*), the volume fraction of fluid ϕ*f*(*z, t*), the fluid velocity in the z-direction $v_{z}^{f}\left(z,t\right)$, the pore pressure in the basement membrane *p*(*z, t*) and the principal effective Cauchy stress $\sigma_{y}^{e}\left(z,t\right)$ (i.e., a measure of the force per unit area acting on a surface element in the deformed BM); *t* is time and *z* is the position along the z-axis. The full derivation of this lubrication model of the poroelastic BM is given in Aldea (2017). However, in section 2.1.2, we provide a more intuitive derivation of this model based on the physiology of the BM system. The governing equations are:

(1) $$\frac{\partial (\phi^{f}h)}{\partial t}+\frac{\partial }{\partial z}(\phi^{f}v_{z}^{f}h)=0,\;$$

(2) $$-\frac{\partial \phi^{f}}{\partial t}+(1-\phi^{f})\frac{1}{h}\frac{\partial h}{\partial t}=0,\;$$

(3) $$\phi^{f}v_{z}^{f}=-\frac{k(h)}{\eta }\frac{\partial p}{\partial z},\;$$

(4) $$\sigma_{y}^{e}-p=\Sigma (z,t),\;$$

(5) $$\sigma_{y}^{e}=f\left(\frac{h}{H}\right),$$

where η is the fluid viscosity and *k* is the deformation dependent permeability of the porous medium (details in Equation 6). *H* denotes the undeformed thickness of the upper half BM and Σ denotes the external constrictive stress; these two terms are the input of the BM model. The function $f\left(\frac{h}{H}\right)$ relates the stress in the BM to its deformation and is derived from a given strain energy function. The reader is referred forward to Equations (8–9) for the particular forms of the stress-strain relationship and the strain energy function used in this work.

#### 2.1.2. Physiological Interpretation of the System (1–5)

The physiological significance of the BM model is outlined below (Aldea, 2017).

Equations (1, 2) represent conservation of fluid and solid mass, respectively, in the deformed configuration of the system. Assuming that the BM is comprised only of fluid and solid phases, the volume fractions ϕ*s* (solid) and ϕ*f* (fluid) satisfy ϕ*s* + ϕ*f* = 1.

Equation (3) is the lubrication approximation of Darcy's law which relates the interstitial fluid velocity to the pore pressure gradient, the fluid viscosity and the deformation-dependent permeability. Various functions for deformation-dependent permeability have been discussed in the literature and, here, we choose the model described in Markert (2005) which stands for a large range of material compression, as well as distension,

(6) $$k(h)=k_{*}{\left(\frac{\frac{h}{H}-\phi_{*}^{s}}{1-\phi_{*}^{s}}\right)}^{\kappa },$$

where k_*_ is the permeability of the undeformed BM, κ is a positive parameter and $\phi_{*}^{s}$ is the volume fraction of the solid in the fluid-filled reference configuration.

Applying an asymptotic analysis (details in Aldea, 2017) shows that, at leading order, the only dimension of the BM that changes significantly is its thickness, meaning that the term $\frac{h}{H}$ represents the Jacobian of the system which gives the change in the volume of the BM. Equations (3, 6) account for the fact that any change in the volume of the BM will affect its porosity and, subsequently, its permeability to fluid flow. The state of zero porosity is reached when $\frac{h}{H}=\phi_{*}^{s}$, meaning that the BM pores are fully closed.

Equation (4) represents the force-balance equation derived from the conservation of momentum. A close look at the elasticity of an arterial segment shows that Σ is the radial stress at the radial position of the BM (see Figure 2, Supplementary Material 1, and Aldea, 2017), so Equation (4) follows from the continuity of radial stress across the BM. In Supplementary Figures 1, 2, we plot the dependence of the radial stress at the middle of the wall on the magnitude of VSMC activation, i.e., Σ(*S*), where *S* acts as a parameter describing the local level of contractile activity of VSMCs. Here, we model the propagation of the muscular contractions of the VSMCs with the following wave form of the vascular tone

(7) $$S(z,t)=A\cdot \sin^{2}\left(\frac{\pi }{\lambda_{w}}\left(z-c_{w}t\right)\right)\cdot S_{m},$$

which was previously used for the muscular activation wave of the ureter wall by Carew and Pedley (1997). For the purposes of this study, the vascular tone wave *S*(*z, t*) represents the vasomotion wave from the V-IPAD model, having the same pattern as the arterial vasomotion wave observed experimentally (Bouskela and Grampp, 1992; Mayhew et al., 1996; Haddock and Hill, 2005; Vetri et al., 2007; Rayshubskiy et al., 2014). *S*_*m*_ reflects the physically observed maximal contraction of the VSMCs (Rachev and Hayashi, 1999) and *A* is the amplitude of change in the maximal muscular contraction. The magnitude of *S*_*m*_ has not been experimentally confirmed in the cerebral muscular arteries. Hence, *S*_*m*_ is taken in accordance with the value reported in carotid arteries (Rachev and Hayashi, 1999). λ_*w*_ and *c*_*w*_ denote the wavelength and the speed of the vasomotion wave, respectively; *t* is time and *z* is the spatial coordinate. The wavelength λ_*w*_ of cerebral vasomotion has not been experimentally measured, but can be calculated by dividing the wave velocity *c*_*w*_ by the wave frequency (or equally by multiplying the wave velocity with the time period of the vasomotion wave). The propagation velocities of the vasomotor response available in the literature describe the induced vasomotor response of arteries, rather than the (spontaneous) vasomotion wave (Duling and Berne, 1970; Dietrich et al., 1996; Seppey et al., 2009).

Finally, Equation (5) represents the constitutive equation, i.e., the stress-strain relationship for the hyperelastic BM. This means that the principal effective Cauchy stress $\sigma_{y}^{e}$ is derived from a strain energy function (here denoted *W*_*BM*_) that describes the elastic behavior of the material under particular deforming conditions. Given the geometrical setup of the BM model, in which compressive forces are only significant in the y-direction, the relationship between the principal stress in the y-direction and the corresponding deformation is given (in terms of a strain energy function) by

(8) $$\sigma_{y}^{e}(h)=f\left(\;\frac{h}{H}\right)=\frac{\partial W_{BM}(\frac{h}{H})}{\partial \lambda_{y}(\frac{h}{H})}.$$

**Figure 3:**
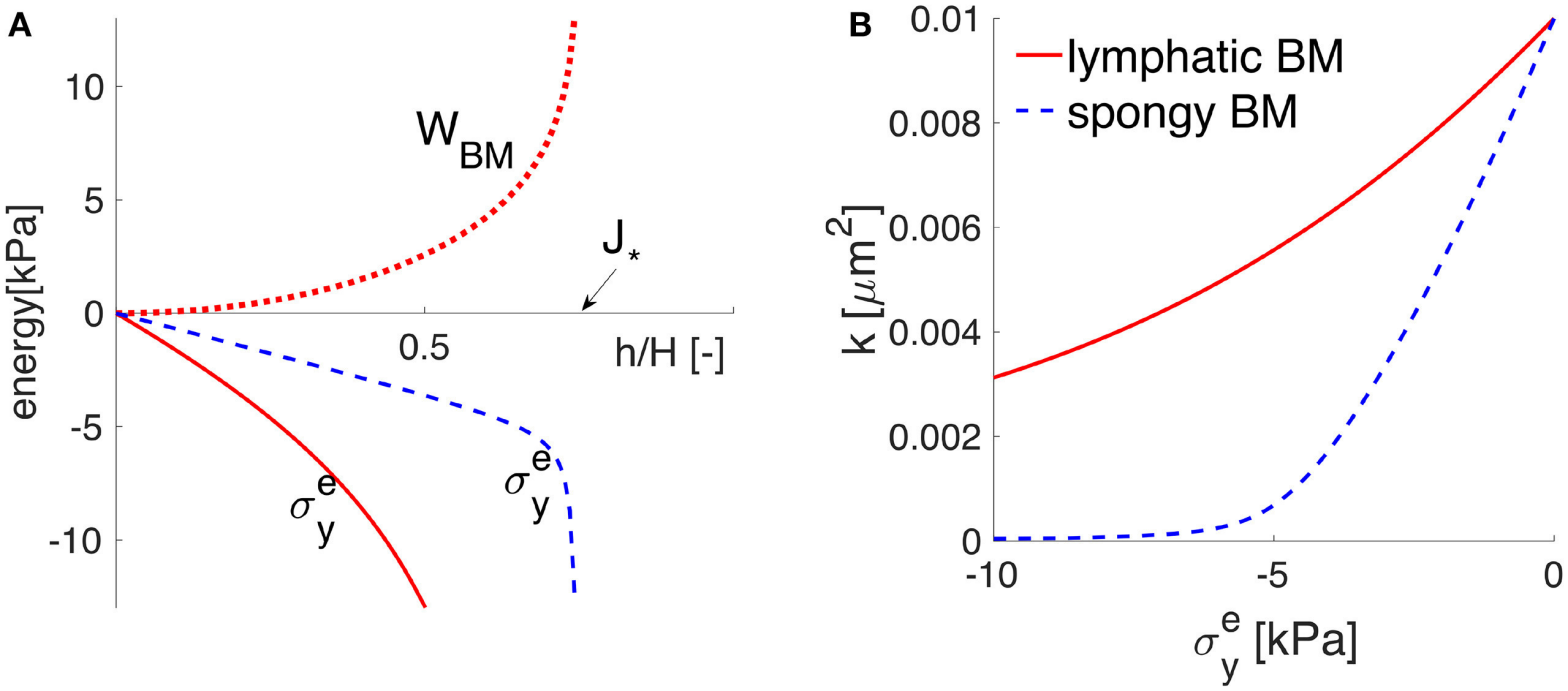
The elastic response of the poroelastic BM. The potential behavior of an idealized “spongy” material is sketched in dotted blue line, while that of the “lymphatic” BM considered throughout this study is plotted in solid red line. **(A)** The strain energy function *W*_*BM*_ = *W*_*BM*_(*h*/*H*) in the upper (positive) part and the principal Cauchy stress $\sigma_{e}^{y}=\sigma_{e}^{y}\left(h/H\right)$ in the lower (negative) part, as functions of the stretch *h*/*H*. The values of *h*/*H* (smaller than one) show that the BM thickness is compressed by the normal elastic stress $\sigma_{e}^{y}$ until reaching the physically-valid lower limit J_*_. **(B)** the stress-permeability relationship, according to which the BM permeability *k* decreases non-linearly with an increases in the compressive normal stress $\sigma_{e}^{y}$.

Here, λ_*y*_ is the principal stretch ratio in the y-direction and is defined as $\lambda_{y}\left(\frac{h}{H}\right)=\frac{h}{H}$, considering that the only dimension of the BM that changes significantly is its thickness. The choice of *W*_*BM*_ requires careful attention. Limited by the lack of experimental data on the elasticity of the cerebrovascular BM, a minimum number of material parameters able to describe the physiological response is desired. With this in mind, we choose the neo-Hookean model with only two material parameters from the study of Wirth et al. (2010), in which various permeability functions (e.g., Equation 6) and hyperelasticity constitutive laws were combined for modeling choked (or limited) flows through biological poroelastic materials. Hence, within this lubrication model, the elastic response of the poroelastic BM to the external constrictive stress is captured by the strain energy function

(9) $$\begin{array}{l} W_{BM}\left(\frac{h}{H}\right)=\frac{\mu_{s}}{2}\left({\left(\frac{h}{H}\right)}^{2}-1-2(1-J_{*})\log\frac{\frac{h}{H}-J_{*}}{1-J_{*}}\right)\;\\ \;+\frac{1}{2}\left(\lambda_{s}-\mu_{s}\frac{J_{*}}{1-J_{*}}\right){\left(\frac{h}{H}-1\right)}^{2}, \end{array}$$

which is plotted in Figure 3. The material parameters μ_*s*_ and λ_*s*_ denote the first and second Lamé parameters, respectively. *H*·J_*_ represents the minimum possible deformed thickness *h* of the membrane. For example, if the solid matrix of proteins is also incompressible, J_*_ equals $\phi_{*}^{s}$ when all the pore spaces are closed. In other words, J_*_ represents the lower limit of physical validity of the employed elastic model, meaning that the BM cannot be compressed to zero volume with finite energy (*W*_*BM*_ diverges as $\frac{h}{H}\to J_{*}$). For the Lamé parameters given in Table 1, the BM behavior is denoted by the word “lymphatic” and the corresponding *W*_*BM*_ from Equation (9) is plotted in Figure 3, showing that it takes a fast increasing energy to deform the BM once its pores are nearly shut (as $\frac{h}{H}$ approaches J_*_). The Cauchy stress $\sigma_{y}^{e}$ is obtained according to Equation (8) and shown in Figure 3. If the BM were a more deformable material (denoted “spongy”), then its pores could be nearly shut during the maximum activation of the VSMCs, e.g., by a compressive stress of 6 kPa (Figure 3 and details in section 4.2).

#### 2.1.3. Concise Formulation of the BM Model

The system of Equations (1–5) is simplified by replacing Equations (2, 3) in Equation (1), which yields the non-linear equation for the deformed upper-half BM

(10) $$\frac{\partial h}{\partial t}=\frac{\partial }{\partial z}\left(h\frac{k(h)}{\eta }\frac{\partial p(h,\Sigma (z,t))}{\partial z}\right),$$

recalling that *k*(*h*) is given by Equation (6) and $p=\sigma_{y}^{e}\left(h\left(z,t\right)\right)-\Sigma \left(S\left(z,t\right)\right)$. Equation (10) requires one initial condition and two boundary conditions.

The BM is considered initially undeformed and uniform along the vessel, i.e.,

(11) $$h{|}_{t=0}=H,\;0\leq z\leq L_{s},$$

where *z* = 0 represents the proximal end of the BM, while *z* = *L*_*s*_ represents the distal end of the BM. Subsequently, both the deformation of the BM induced by the external compressive stress and the resulting flow through the BM, are investigated.

The ends of the BM are assumed to be at the same pressure (i.e., the pressure drop along the BM between the two ends of the vessel does not drive a significant flow)

(12) $$p{|}_{z=0}=p{|}_{z=L_{s}}=0,\;t>0.$$

Equation (10) plays a paramount role in the dynamics of the system and is solved for *h*(*z, t*) for a given Σ(*S*(*z, t*)) and *W*_*BM*_. Once *h* is determined, all the other variables of the system can be calculated given their dependency on *h*. Finally, the fluid flow rate through the poroelastic BM is calculated as shown below.

#### 2.1.4. Volumetric Flow Rate Through the Poroelastic BM

An infinitesimal element of the cerebral artery is considered, as illustrated in Figure 2. The annular region represents the poroelastic BM. Radial symmetric deformation of the artery is assumed and the deformation of BM depends solely on the external compressive stress induced by the vasomotion wave, i.e., on Σ(*S*(*z, t*)). The total flow rate of fluid through the infinitesimal volume of BM, generated by the vasomotion-induced deformation of the BM boundaries, is calculated by using the differential form of the Darcy's law and recalling that the cross sectional area of the BM is $2\pi r_{m}\left(2h\right)\left[\mu m^{2}\right]$; hence, it follows that

(13) $$Q_{BM}(z,t)=-2\frac{2\pi r_{m}hk(h)}{\eta }\frac{\partial p}{\partial z},$$

where *Q*_*BM*_(*z, t*)[μm^3^·s^−1^] is the volumetric flow rate through the entire thickness of the BM and *r*_*m*_[μ*m*] denotes the radial position of the BM. Here, the extra factor of 2 appears because we have only been considering the upper half of the BM in our model from sections 2.1.1–2.1.3. It is emphasized that *r*_*m*_ is a function of the wave of vascular tone, i.e., *r*_*m*_ = *r*_*m*_(*S*(*z, t*)), and its form is not prescribed, but is rather determined from the arterial wall model in Supplementary Material 1.

The net flow rate over one cycle of vasomotion is

(14) $$\begin{array}{ll} {\bar{Q}}_{BM} & =\frac{1}{T}\int_{0}^{T}Q_{BM}dt, \end{array}$$

where *T* represents the time period of the vasomotion wave. The parabolic Equation (10) is solved with the method of lines as described in Supplementary Material 2. All the common diagnostic checks were performed to ensure that the numerical method conserves the mass of the system and the errors are acceptably small. The convergence of the employed numerical method is demonstrated in Supplementary Figure 3.

## 3. Results

We have considered the cerebral artery to be an idealized vessel (e.g., thick-walled cylinder) with uniform material properties determined experimentally by Bell et al. (2013). For reasons of simplicity, we have accounted for a single layer of BM positioned at the middle of the arterial wall and modeled it as a fluid-filled poroelastic material with a slow-varying thickness, as illustrated in Figure 2. We have assumed the BM boundaries to be impermeable, such that the fluid cannot escape from this compartment, guaranteeing in this way conservation of fluid. The artery deforms due to the non-zero intraluminal pressure and longitudinal stretching, as well as due to VSMC contractions (i.e., active mechanical response). The biomechanical behavior of three MCAs was investigated in detail in Supplementary Material 1 and the results corresponding to an artery exposed to a physiological intraluminal pressure of 13.3 kPa and an axial stretch of 1.07 are presented below. The deformation of the artery wall triggers the flow in the BM, but the BM is small enough such that it does not alter the elastic stresses in the wall. Hence, there is only one-way coupling, allowing for the elastic analysis of the artery to be handled independently of the BM.

Rather than prescribing the deformation of the arterial wall, we have first solved the arterial wall model from Supplementary Material 1 in order to assess the variations in arterial diameter as a consequence of the local VSMC contractions, by maintaining both the intraluminal pressure and the axial stretch constant. Once the deformed inner radius of the artery is determined, the deformation and the corresponding stresses at any spatial point within the wall can be calculated. Secondly, by assuming continuity of stress across the wall, we can take the radial stress (denoted σ_*r*_) at the middle of the arterial wall as the external constrictive stress (denoted Σ) acting on the BM, i.e., σ_*r*_(*r* = *r*_*m*_) = Σ, where *r*_*m*_ is the deformed radius *r* at the middle of the arterial wall (see Figure 2 and Supplementary Figure 1).

**Figure 4:**
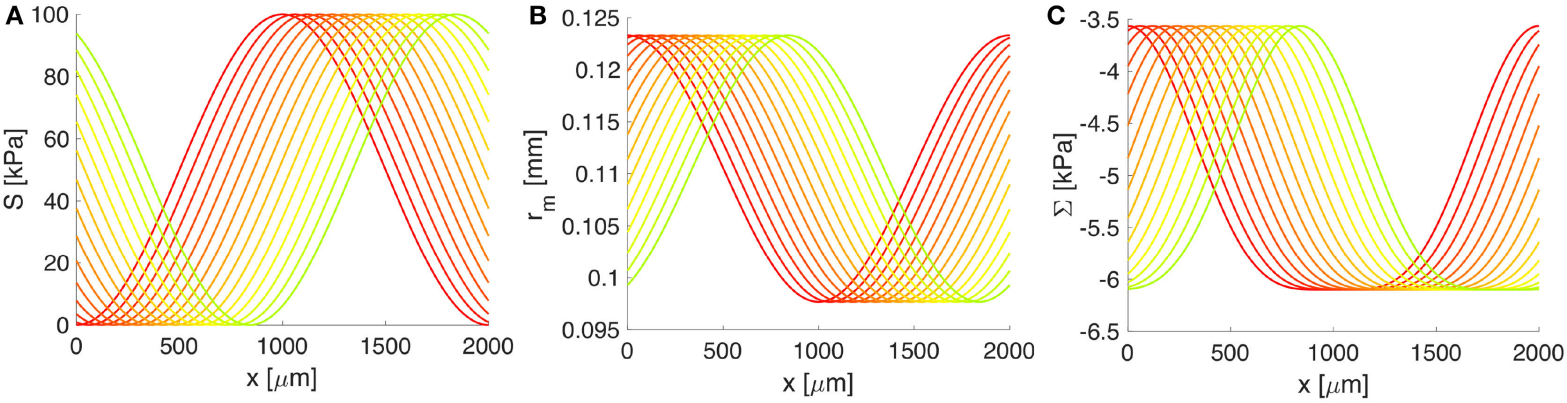
The response of the artery wall to muscular oscillations over one wavelength. **(A)** The vasomotion wave, **(B)** the radial position of the BM, and **(C)** the constrictive stress acting on the BM. Both *r*_*m*_ and Σ depend on *S*(*z, t*) and serve as input in the BM model. Progressive change from the red to the green curve shows increase in time; only 4 s from one vasomotion cycle of 10 s are illustrated.

Furthermore, the spatiotemporal contractile oscillations of the cerebral VSMCs, i.e., the vasomotion wave, are prescribed by the wave form of the vascular tone *S*(*z, t*) with units of stress, as shown in Figure 4 and defined in Equation (7). The values of *S* were determined elsewhere (Rachev and Hayashi, 1999) based on experimentally recorded pressure-diameter curves of arteries, under VSMC contraction and control conditions. Thereby, *S* = 0 kPa reflects the purely passive mechanical response of the arterial wall when the VSMCs are fully relaxed, while *S* = 100 kPa is taken for maximal muscular contraction. In terms of spatiotemporal properties of the system, we consider the frequency of vascular oscillations to be around 0.1 Hz (i.e., they repeat every 10 s) and investigate the particular case in which the wavelength of the vasomotion wave is comparable with the characteristic length of a MCA. Moreover, for reasons of simplicity, we assume a uniform system and one that is sufficiently long in order to encompass several vasomotions waves simultaneously (see Table 1). This latter consideration allows for evaluation of the solution to our poroelastic problem far from the ends of the computational spatial grid where rapid variations in the solution may occur.

Both the deformation of the middle wall of the artery, *r*_*m*_ = *r*_*m*_(*S*(*z, t*)), and the corresponding radial stress, Σ = Σ(*S*(*z, t*)), depend on the vascular tone *S*(*z, t*) through the arterial wall model (see details in Supplementary Material 1). In turn, these quantities serve as input in the BM model (see Figure 4). A whole cycle of vasomotion includes both the contraction and the relaxation of VSMCs and the corresponding oscillation between the maximal and minimal radius of the artery has an amplitude of approximately 20%, as pictured in Figure 4. In addition, from Figure 4, it is obvious that the highest negative value of Σ (i.e., the strongest constrictive stress on the BM) is generated during maximal muscular contraction. On the other hand, the lowest value of Σ corresponds to the case of fully relaxed VSMCs and is non-zero due to stresses generated by the passive load bearing components of the wall of the artery (e.g., collagen and elastin fibers) during inflation and axial stretching of the artery.

### 3.1. The VSMCs as the Pump for IPAD in the Brain

The spatiotemporal variations in cerebral vascular tone influence the fluid flow through the BM by inducing deformations of its top and bottom boundaries via the radial constrictive stress Σ(*S*(*z, t*)). The fluid movement through the BM is governed by Darcy's law. The change in the BM volume affects its porosity and, subsequently, its permeability to fluid flow, according to Equations (3, 6). In this particular scenario, it has been allowed for the fluid volume fraction to drop to zero due to finite compressive forces that reduce the BM pores and, as a consequence, obstruct the path for fluid drainage; this behavior is similar to squeezing shut a fluid-filled sponge.

**Figure 5:**
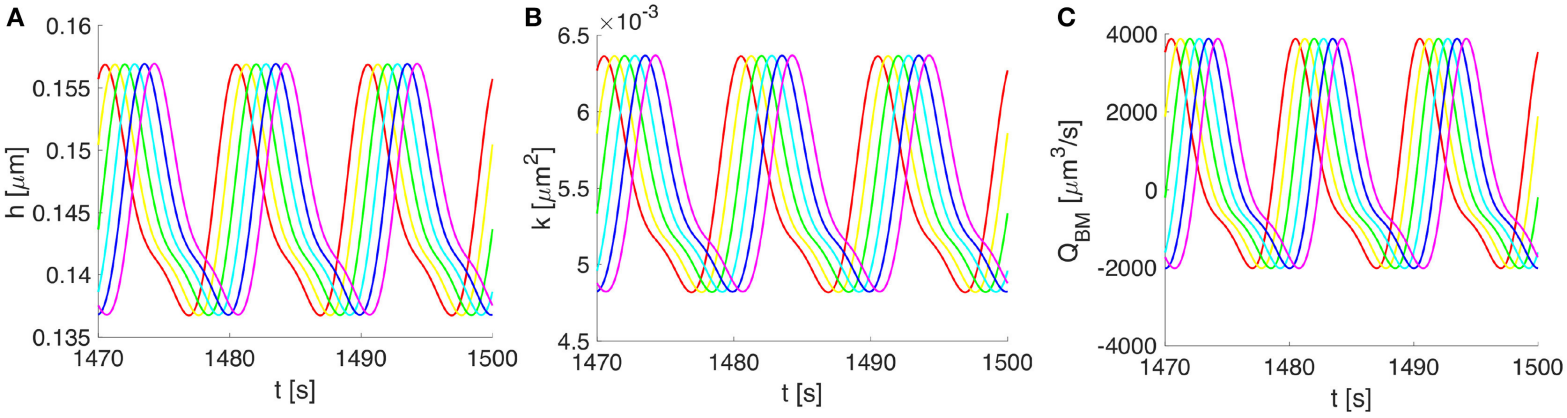
Vasomotion-induced deformation and flows along the BM for three cycles of vasomotion. **(A)** Oscillations in time of the upper-half thickness of the BM, **(B)** deformation dependent permeability of the BM, and **(C)** the corresponding volumetric flow rate along the whole compartment of BM. Progressive change in color from red to magenta shows six distinct spatial points over 750 μm (out of a 2,000 μm wavelength).

The response of the poroelastic BM to the external constrictive stress is described by the stress-stretch relationship from Equation (8), which is derived using the strain energy function for neo-Hookean poroelastic materials from Wirth et al. (2010). The ability of the chosen strain energy function from Equation (9) to describe a non-linear elastic behavior for a general large range of deformations is shown in Figure 3 and further discussed in section 4.2. The cerebrovascular BM considered here has similar properties to the interstitium of the systemic lymphatic system (Smillie et al., 2005) and to the interstitium of the brain (Heppell et al., 2013), therefore it appears as a physiologically reasonable assumption. From Figures 3, 5, it is obvious that the compressive stress generated during maximum VSMC activation (e.g., 6 kPa) decreases the permeability of the BM by 50% compared to its undeformed state. As the BM pores are squeezed by the contractile VSMCs, the reduced BM permeability prevents high reverse flows (in the direction opposing the vasomotion wave), making the VSMCs an efficient pump for driving fluid along the IPAD pathways.

### 3.2. Non-zero Net Intramural Periarterial Flow Rates During One Cycle of Vasomotion

Owing to the assumed symmetry of the system pictured in Figure 2, the vasomotion-induced BM deformation has been determined by solving Equation (10) for the upper-half part of the BM. The BM deformations propagate longitudinally and vary in time, with characteristics specific to the vasomotion wave (e.g., a time period of 10 s or, equally, a frequency of 0.1 Hz). The evolution of the BM thickness *h*(*z, t*) and permeability *k*(*z, t*), as well as the resulting fluid flow rate *Q*_*BM*_(*z, t*) through the BM are shown in Figure 5. The vasomotion-induced fluid flow rate through the entire BM compartment, calculated with Equation (13), depends on the radial deformation of the artery wall, the fluid viscosity, the deformation-dependent permeability of the poroelastic BM and the pore pressure gradient. Given that one vasomotion cycle incorporates both the contraction and the relaxation of the VSMCs, positive (i.e., in the direction of the vasomotion wave) as well as negative flows (i.e., in the reverse direction of the vasomotion wave) occur at different times at a given spatial point of the BM. Although high intramural flow rates of nearly 4000 μm^3^·s^−1^ are obtained, the net flow rate during one cycle of vasomotion is only 360 μm^3^·s^−1^. The positive value indicates that the net intramural fluid flow rate during one cycle of vasomotion is always in the direction of the vasomotion wave (a consequence of the much reduced BM permeability as it is squeezed; Aldea, 2017).

## 4. Discussion and conclusion

The small dimension of the clearance pathways of the brain (e.g., 10–400 nm) and their anatomical position within the deep cortical layers make the investigation of brain clearance challenging to conduct in living humans and even in animal models. Physiologically-based mathematical and computational models represent an alternative tool for analysing potential clearance mechanisms of the brain, likely generating new mechanistic insights (Goriely et al., 2015; Holter et al., 2017). Since the motive force for IPAD has remained unresolved (Diem et al., 2017), we have proposed and tested with *in silico* modeling the idea of vasomotion-driven IPAD (denoted V-IPAD). According to the V-IPAD hypothesis, the contractile VSMCs of cerebral arteries act as the drivers of IPAD in the brain by inducing BM deformations and, subsequently, net intramural fluid flows in the direction of the vasomotion wave. To our knowledge, this is the first quantitative study that explores the contribution of the cerebral VSMCs in the drainage of fluid from the brain. Although the exact physiological roles of vasomotion remain elusive, the most common view is that vasomotion serves as an auxiliary mechanism in tissue perfusion, especially under hypoperfusion conditions (Nilsson and Aalkjær, 2003). The theoretical results from this study indicate another potential role for vasomotion, namely the clearance of fluid and soluble metabolites from the brain along the IPAD pathways.

### 4.1. Experimental Evidence Supporting the V-IPAD Hypothesis

The most solid evidence for the V-IPAD hypothesis probably comes from the experiments of Beach et al. (2000) who found significant accumulation of Aβ within the abluminal BM of VSMCs as a result of cortical cholinergic deafferentation (i.e., interruption of acetylcholine neurotransmitter to the cerebral blood vessels and other cells of the brain). The authors found it difficult to explain why the Aβ deposition was predominantly in the wall of arterioles and only sparsely in the brain tissue as amyloid plaques. Here, we propose that the cerebrovascular accumulation of Aβ, observed in Beach et al. (2000), resulted from impaired activity of the VSCMs. As the vascular tone of cerebral arteries is modulated by a rich cholinergic innervation (Hamel, 2006), the cholinergic deafferentation induced in the study of Beach et al. (2000) may have hindered the VSMC activity and, subsequently, their contribution to the clearance of Aβ along the BMs that act as the IPAD pathways.

Inefficient periarterial drainage was observed during ischemic stroke (i.e., focally disrupted blood perfusion to the brain) in Arbel-Ornath et al. (2013). Since the contractile filaments of the arterial VSMCs are significantly damaged after 15–45 min of ischemia (Kwon et al., 2002), altered contractile activity of the VSMCs of the vessels deprived of blood flow seems likely and this could explain the impaired periarterial drainage along occluded vessels observed by Arbel-Ornath et al. (2013). On a similar note, Carare et al. (2008) observed no perivascular drainage following cardiac arrest. Further evidence that indicates a link between the cerebral vascular tone and IPAD comes from Maki et al. (2014) where the vasoactive drug, Cilostazol, was administrated into the mouse brain, leading to enhanced periarterial drainage.

### 4.2. The Most Likely Motive Force for IPAD in the Brain

The simulated amplitude of oscillations in arterial diameter (e.g., 20%), induced by the contracting and relaxing VSMCs, is in line with the experimental observations of vasomotion in large cerebral arteries (Fujii et al., 1990, 1991; Rayshubskiy et al., 2014) and is considerably larger than the amplitude of oscillations induced by systolic pulsations (e.g., on average 3%, Bedussi et al., 2018). Comparison of the resulting intramural fluid flow rates with previous studies is given in Table 2. The net fluid flow rate of 360 μm^3^·s^−1^ generated by the V-IPAD mechanism along only one BM layer is five orders of magnitude higher than the net periarterial flow rate induced by arterial pulsations (Diem et al., 2017). The studies of Asgari et al. (2015, 2016) also yielded perivascular flow rates driven by arterial pulsations, but in a distinct perivascular space positioned between the arterial wall and glial layer of the brain. Nonetheless, their findings offer additional proof that arterial pulsations are incapable of generating significant perivascular flow rates out of the brain. The values reported by Asgari et al. (2015, 2016) differ from those reported by Diem et al. (2017) due to the fact that the former authors assumed the thickness of the perivascular space up to two orders of magnitude higher than in the model of Diem et al. (2017). Here, the BM thickness is taken to be 0.4 μm in the undeformed, hydrated state and decreases to 0.27 μm during its compression by the maximally activated VSMCs. It is worth remarking that when comparison with other studies is made, it should be kept in mind that others may have defined the net flows out of the brain as negative flows relative to the direction of arterial pulsations.

**Table 2:** Comparison of results between the V-IPAD model and previous models.

**Study**	**Space**	**Thickness**	**Net flow rate**
		**[μm]**	**[μm^**3**^ · s^**−1**^]**
V-IPAD model	Poroelastic BM	0.4	360
Diem et al., 2017	Porous BM	0.2	1.12 ·10^−3^
Asgari et al., 2015	PVS	1	2.94 ·10^−2^
Asgari et al., 2016	PVS	10	3.72 ·10^−1^

*The driving force in the V-IPAD model is the vasomotion wave, while in the models of Diem et al. (2017), Asgari et al. (2015), and Asgari et al. (2016) is the arterial pulse. The net flow rates represent the absolute values. BM is the compartment within the arterial wall, while PVS denotes the perivascular space between the arterial wall and the glial layer of the brain*.

Compared with the arterial pulse, the cerebral vasomotion is a more reasonable driving force of IPAD because its wavelength is orders of magnitude smaller than that of the arterial pulse wave. The wavelength of vasomotion is calculated based on its frequency and velocity. While the frequency of vasomotion is commonly reported with values centred at 0.1 Hz, its velocity is less well known. However, existing data allow a reasonable estimate. For example: (i) rat arterial strips elicited vasomotion with a velocity of 100 μm/s (Seppey et al., 2009), while (ii) hemodynamic oscillations propagating on the surface of the human brain (probably related to vasomotion) had an average velocity of 800 μm/s (Noordmans et al., 2018). Even when the higher end of velocities is considered, the resulting wavelength of cerebral vasomotion (e.g., 8 mm) is still two orders of magnitude smaller than that of the human arterial pulse wave (e.g., 1 m, calculated based on a velocity of 1 m/s and a frequency of 1 Hz, Stefanovska, 2007). In particular, vasomotion wavelength appears comparable with the length of the cerebral arteries; this feature favors development of pressure gradients that are able to drive significant net ISF flows along the IPAD pathways.

Despite the fact that the V-IPAD mechanism promotes significantly higher net intramural periarterial flow rates than the previously suggested mechanisms (see Table 2), it is essential to examine the relative contribution of V-IPAD in the global picture of brain clearance. The model we have designed yields the fluid flow rate along only one BM compartment surrounding the arterial VSMCs. The total number of BMs contributing to IPAD in the whole brain is currently unknown, so instead we consider a smaller block of gray matter and its vascular supply which can be relatively easier to assess. Based on the *in vivo* imaging of the surface arterial vascular network in the rodent brain (Schaffer et al., 2006), minimum 8 surface arteries branching from the MCA can be detected in an area measuring 4 × 3 mm. It is reasonable to assume that the penetrating arterioles arising from the surface arterial network extend through the entire depth of the cortex (1 mm in the rat brain Vetreno et al., 2017). In this way, a brain volume measuring 4 × 3 × 1 mm appears to be supplied (blood through the lumen) and cleared (ISF and soluble metabolites through the artery wall) by at least 8 surface arteries branching from the MCA. Assuming that each of these surface arteries has on average 3 BM compartments (Lee, 1995), a total of 24 BMs clears a block of gray matter of 12 mm^3^, leading to a net ISF flow rate of 720 μm^3^·s^−1^·mm^−3^ (calculated as 24 · 360 μm^3^·s^−1^/12 mm^3^) or, equally, 0.04 μl·min^−1^·${\text{g}}_{\text{brain}}^{-1}$. Owing to the lack of consistent experimental data on the passive and active mechanical response of small cerebral arteries, we have considered the vascular response of the branches of MCA to be identical to that of the MCA itself when estimating the total net ISF flow rate drained from a block of gray matter along the IPAD pathways. We note that investigation of IPAD in the MCA is itself an important goal, because the MCAs, together with the Circle of Willis, may be seen as a bottleneck in the outflow of ISF and solutes toward the cervical lymph nodes along the IPAD pathways. Therefore, it is of high significance to evaluate the likely strength of IPAD drainage along the MCAs. The proposed mechanism can also explain vasomotion-driven IPAD in smaller cerebral arteries with at least two layers of VSMCs. Adaptation of the V-IPAD model to intracerebral arterioles with only one layer of VSMCs will be addressed in a future study.

How physiologically-significant is the estimated net ISF flow rate along the IPAD pathways? The exact amount of ISF that requires removing from the brain interstitium along the IPAD pathways vs. other routes (e.g., convection through the cerebral extracellular spaces toward the CSF Abbott, 2004 or the recently proposed glymphatic system; Iliff et al., 2012) has been widely debated (Hladky and Barrand, 2014). By comparison with the average value for ISF flow rate from the brain (e.g., 0.2 μl·min^−1^·${\text{g}}_{\text{brain}}^{-1}$), which falls within the range of lymph flow rates and was estimated by Szentistvanyi et al. (1984), our estimated fluid flow rate induced by the V-IPAD mechanism (e.g., 0.04 μl·min^−1^·${\text{g}}_{\text{brain}}^{-1}$) has a 20% contribution to the clearance of cerebral ISF secreted at the blood-brain barrier. The amount of ISF transported by the V-IPAD mechanism may prove sufficient to maintain a physiological Aβ concentration within the artery wall by diluting the soluble Aβ that is not cleared at the blood-brain barrier via LRP1 transporters (escaping instead along the BM of cerebral arteries).

The elastic properties of the BM and the pattern of the vasomotion wave prove crucial in inducing net intramural fluid flow rates of physiological importance. If the cerebrovascular BM has the purpose of removing most of the cerebral soluble metabolites, its properties will likely have evolved to improve clearance of the brain and, hence, the BM may be a more deformable material than the one simulated here. The behavior of the BM may resemble the elastic response of the “spongy” material illustrated in Figure 3, which could undergo more significant compression of its pores, thereby hindering almost completely the reverse intramural flow. Such a behavior should make the pumping action of cerebral VSMCs even more effective, leading to higher net intramural fluid flow rates than the ones simulated here. This encourages future experimental assessment of the biomechanics of the contractile cerebral VSMCs and their BMs in order to explore the full potential of the V-IPAD mechanism. In the light of the values given in Table 2, the vasomotion wave appears as the best candidate proposed to date for the motive force for IPAD in the brain because it has a more appropriate amplitude and wavelength to drive fluid out of the brain at physiologically more significant flow rates than the arterial pulse. This conclusion holds for all the arterial segments described in Supplementary Material 1 (with material parameters in Supplementary Table 2), considering that the resulting net intramural flow rates are 380 μm^3^ · s^−1^ and 444 μm^3^ · s^−1^ when the artery experiences an axial stretch of 1.09 and 1.13, respectively.

### 4.3. The Direction of the V-IPAD Mechanism

We have shown that the oscillations in the contractile state of the cerebral VSMCs are sufficiently powerful to promote net intramural flow rates of notable magnitude for efficient drainage of fluid along the IPAD routes, but the predominant direction of vasomotion remains elusive. Within the context of IPAD of the brain, the vasomotion wave must propagate from penetrating arterioles toward leptomeningeal arteries on the surface of the cortex. Direct investigations of the cerebral cortex in humans when awake showed distinct regions of pial arterioles exhibiting slow sinusoidal vascular oscillations, with a frequency of ≈ 0.1 Hz, which propagated as spatial waves across the cortex (Rayshubskiy et al., 2014). Spontaneous, low-frequency oscillations of cerebral arteries within the range 0.1–1 Hz, presumably resulting from vasomotion, were also reported in mice when awake, propagating over several hundred micrometers across the cortex (Drew et al., 2011). Remote communication of local vascular responses (e.g., vasodilation) of arterioles to their parent arteries is common during cerebral functional hyperemia, in order to assure an optimal blood supply to the highly active brain region (Iadecola et al., 1997). Considering the complexity of communication between the active neurons and their supplying blood vessels during cerebral functional hyperemia, the cerebral arterial network seems to be empowered with properties that allow vasomotion to propagate in the optimal direction for IPAD (Secomb and Pries, 2002; Girouard and Iadecola, 2006; Sweeney et al., 2018). Additional experimental recordings of the spatial pattern of vasomotion is required and *in vivo* optical imaging (e.g., two-photon microscopy, optical intrinsic signal imaging) of contractile cerebral vascular networks (Nishimura et al., 2007; Chen et al., 2011; Rayshubskiy et al., 2014) will prove useful in such an endeavor.

It has recently been shown that the permeability of the brain interstitium is too low to allow development of any substantial bulk flow at physiological intracerebral pressure differences (Jin et al., 2016; Holter et al., 2017). Soluble metabolites can thus only leave the brain tissue by either diffusion toward the CSF compartments or by bulk flow along less resistant pathways such as the perivascular pathways of blood vessels (Abbott, 2004; Bakker et al., 2016). However, diffusion is only able to operate effectively over very short distances (Syková and Nicholson, 2008). It is not immediately obvious how the vasomotion wave will improve the flow of fluid and soluble metabolites along the other proposed para-vascular clearance pathways, such as the pial-glial basement membranes (Albargothy et al., 2018) or the para-venous pathways (Iliff et al., 2012). According to the recent review of Welsh et al. (2018), there is no evidence of conducted vasomotor responses along venules and veins, or from veins to the arterial system through the capillary network. This indicates that vasomotion can only act as a driving force for periarterial drainage. Furthermore, if the artery wall acts as a two-way traffic system for (i) the efflux of ISF and soluble parenchymal metabolites along the IPAD pathways and (ii) the convective influx of CSF into the brain parenchyma along the pial-glial basement membranes, then two distinct motive forces are required. With respect to the latter, it has been demonstrated by Asgari et al. (2016) that the arterial pulsations propagating into the brain are not sufficient to drive significant net para-vascular flows along arteries, but they may enhance local mixing and diffusion of solutes in the CSF along para-vascular pathways. It remains an open question which exact physical forces are responsible for this influx of CSF into the brain parenchyma. In order for separate longitudinal flows (in opposite directions) to occur along both the IPAD and para-vascular pathways, the vascular BM, adjacent to the VSMCs, needs to be separated from the CSF by an impermeable (or highly resistant) membrane. The pial layer could serve this purpose, but its exact permeability needs further experimental characterization, as it may not only vary between species, but also between intracerebral and extracerebral regions (Alcolado et al., 1988; Abbott et al., 2018). Here, we have shown that vasomotion can drive net ISF flow along the vascular BM positioned between two impermeable layers of VSMCs.

### 4.4. Contributors to the Pathogenesis of CAA

While direct evidence is still missing, the aforementioned experiments (Beach et al., 2000; Arbel-Ornath et al., 2013; Maki et al., 2014) strongly support the proposed hypothesis that the activity of the cerebral VSMCs may be a key element for IPAD in the brain and, subsequently, in preventing the onset of CAA. The contractile and relaxing abilities of the VSMCs are weakened in old arteries (Tümer et al., 2014). The cholinergic innervation of cerebral arteries also decreases with age and in the presence of AD, thus impairing vessel dilatation (Tong and Hamel, 1999; Van Beek and Claassen, 2011). Moreover, the transport of Aβ out of the brain interstitium across the blood-brain barrier also becomes inefficient with age (Shibata et al., 2000; Deane et al., 2009), leading to an elevated burden of intracerebral Aβ that requires removal along alternative routes, presumably along the IPAD pathways. The cumulative effect of all these age-driven changes may translate into an altered response of the VSMCs and, consequently, into a diminished motive force for IPAD, increasing in this way the likelihood of CAA.

Various hypotheses have been advanced to explain CAA. It has been proposed that the Aβ deposited in the walls of arteries in CAA was derived from VSMCs (Vinters, 1987). However, production of Aβ by VSMCs does not explain the deposition of Aβ in the walls of capillaries that lack VSMCs. Furthermore, transgenic mice in which neurons overproduce Aβ develop CAA, both in cortical and leptomeningeal arteries; this suggests that parenchymal Aβ is transported from the brain tissue along the walls of arteries where it accumulates due to amyloid overload of the drainage pathways (Calhoun et al., 1999). It has also been proposed that the origin of Aβ in the walls of arteries in CAA is from the CSF due to convective influx along para-arterial pathways (Iliff et al., 2012). However, subsequent studies have shown that tracers from the CSF enter the brain along pial-glial basement membranes (Morris et al., 2016) and drain from the brain along the IPAD pathways (Albargothy et al., 2018). The most feasible mechanism for the pathogenesis of CAA, therefore, appears to be protein elimination failure angiopathy due to age-related failure of IPAD (Carare et al., 2013).

### 4.5. Potential Therapeutic Implications

The impact of this study opens avenues for targeting vasomotion toward the facilitation of IPAD and prevention of CAA. This is key for the successful outcome of current immunization trials against Aβ in which plaques are solubilized, but Aβ becomes entrapped in the IPAD pathways, increasing the severity of CAA and most likely resulting in amyloid-related imaging abnormalities (Holmes et al., 2008; Sperling et al., 2012; Weller et al., 2015). Moreover, as neurovascular therapeutic approaches in patients with mild cognitive impairment and AD are developed, e.g., the clinical trial with Cilostazol (Saito et al., 2016), the potential role of cerebral VSMCs in the periarterial clearance of Aβ from the brain may be worth considering in greater detail. The role of the VSMCs in the efficiency of IPAD in the brain could be tested experimentally in animal models with endogenous dysfunction of the VSCMs, which could be induced by dietary interventions (Gooch and Wilcock, 2016) or, in a more specific manner, by genetic mutations (Lee et al., 2012).

Using transgenic models with overproduction of mutant amyloid-beta peptides has many advantages in basic understanding of mechanism of disease (Klohs et al., 2014) but has also led to problems regarding translation into therapies (Jäkel et al., 2017). One model that may be more suitable, as there are no transgenic manipulations, is that of HHcy (Gooch and Wilcock, 2016). The HHcy mouse model develops physiologically relevant cerebrovascular pathology including microhemorrhages, vascular fibrosis, cerebral hypoperfusion and, most importantly, cognitive impairment (Hainsworth et al., 2016; Sudduth et al., 2017). The HHcy model is a dietary intervention that drives the folate cycle and methionine metabolic pathways to accumulate homocysteine through the elimination of vitamins B6, 9, and 12, and enrichment with methionine. The levels of homocysteine achieved in this model equate to moderate HHcy for a mouse. Our pilot data from HHcy models presented in Supplementary Material 3 show a strong trend of reduced expression of α-smooth muscle actin in the HHcy mouse model compared to the control group (see Supplementary Figure 4). This suggests that the normal contractile function of the VSMCs may be impaired in the HHcy cases. In the future stages of our experiments, we will investigate the efficiency of IPAD in the HHcy mouse model.

Drugs such as cholinesterase inhibitors, commonly prescribed in the treatment of Alzheimer's disease, have enhanced high cognitive functions in demented patients who responded to treatment (Birks, 2006). Under the cholinergic-vasculature hypothesis, Claassen and Jansen (2006) have proposed that the observed benefits may be an outcome of the direct action of cholinesterase inhibitors on blood vessels. It therefore remains an interesting question how various vasoactive agents acting on the cerebral VSMCs affect not only the cerebral blood flow, but also the perivascular drainage of Aβ out of the brain.

In conclusion, this study has theoretically proven that the vasomotion wave initiated by the contractile VSMCs of cerebral arteries can represent the motive force for IPAD in the brain, shifting the view from a heart-driven clearance of fluid and solutes from the brain to an intrinsic mechanism of cerebral arteries. The V-IPAD model is the first one that quantitatively examines the contribution of the contractile cerebral VSMCs in the clearance of fluid from the brain, proposing a physically plausible driving force for IPAD. The model offers mechanistic insights into the behavior of the cerebrovascular BM and its interaction with the adjacent VSMCs. Once additional experimental data about the active response of cerebral arteries are provided, the V-IPAD model could be extended in order to improve its accuracy and simulate the effect of cerebral vascular aging on the IPAD of the brain. The hypothesis proposed and tested here stimulates further experimental investigation of the contribution of cerebral VSMCs in the clearance of fluid and solutes from the brain, particularly as these cells represent an accessible therapeutic target for CAA.

## Ethics Statement

The study was approved by the University of Kentucky Institutional Animal Care and Use Committee and conformed to the National Institutes of Health's Guide for the Care and Use of Laboratory Animals.

## Author Contributions

RA, GR, and RC formulated the hypothesis. RA and GR formulated and solved the model. RC and DW designed the experimental pilot study, obtained, and analyzed the experimental data. All authors contributed to the draft of the manuscript, read, and approved the final version.

### Conflict of Interest Statement

The authors declare that the research was conducted in the absence of any commercial or financial relationships that could be construed as a potential conflict of interest.
