# Supplementary material for: Cerebrovascular Smooth Muscle Cells as the Drivers of Intramural Periarterial Drainage of the Brain
Source: Front Aging Neurosci. 2019 Jan 23;11:1. doi: 10.3389/fnagi.2019.00001 (PMC6357927; doi:10.3389/fnagi.2019.00001)
Supplement: Supplementary file 1 [file Data_Sheet_2.PDF]

Supplementary Material:
Cerebrovascular smooth muscle cells as the drivers of intramural periarterial drainage of the brain

Roxana Aldea, Roy O. Weller, Donna M. Wilcock, Roxana O. Carare * and Giles Richardson
*Correspondence:
Roxana O. Carare
R.O.Carare@soton.ac.uk

1 ARTERIAL WALL MODEL

The arterial wall model used to calculate the radial position of the deformed basement membrane (BM) (denoted r_m) and the corresponding stresses (denoted Σ) is given below. An active cerebral artery is described in cylindrical coordinates as a long, elastic thick-walled cylinder exposed to radial inflation and longitudinal extension. The artery wall is a continuous, deformable material such that the stress response to the imposed forces induces deformations. The undeformed (reference) configuration is given in terms of (R, Θ, Z) in the Lagrangian (material) frame. The artery wall is deformed in such a way that, at a later time, the position $\mathbf{X}$ of an arbitrary material point is displaced to a new position $\mathbf{x}(\mathbf{X}, t)$, where t denotes time and $\mathbf{x}$ denotes the position vector in the deformed (current) configuration described in terms of (r, θ, z) in the Eulerian (spatial) frame. The arterial wall deformation is captured by the deformation gradient tensor (here denoted $\mathbf{F}^w$) calculated as the $F^w_{ij} = \frac{\partial x_i}{\partial X_i}$ (with $i, j = r, \theta, z$) (Kalita and Schaefer, 2008).

The arterial wall model is in line with the experimental investigation of rat middle cerebral arteries from Bell et al. (2013). Three non-contracting arteries, i.e. with fully relaxed vascular smooth muscle cells (VSMCs), are inflated with an intramural pressure P of 13.3 kPa and maintained at a constant axial stretch (here denoted λ_ζ), describing in this way a passive mechanical response (Bell et al., 2013). We simulate additional deformations of the arteries due to the contraction of VSMCs, thereby accounting also for the active mechanical response of the arteries. The reference state is considered to be the zero-load state (i.e. the intraluminal pressure and the axial strain are both zero and the VSMCs are fully relaxed) with a mean undeformed inner radius R_i of 0.085 mm and a mean underformed outer radius R_o of 0.125 mm.

The wall deformation and stresses are calculated under the assumption that the deformation of the artery is axisymmetric (no θ-dependence) and weak in the axial direction of the artery. Consequently, the deformation can be approximated by a quasi one-dimensional solution in which the dependent variable is the r (radial) coordinate, while the z (axial) coordinate has the role of a parameter. Moreover, no time-dependence is considered. The artery wall is assumed incompressible (i.e. the volume of the artery wall is preserved during deformation). Given that the incompressible artery only experiences radial inflation and longitudinal extension, i.e. no torsion, it is acceptable to further simplify the problem by considering the axes of the cylindrical coordinate system oriented along the principal directions of the Cauchy stress, meaning that the only non-zero physical components of the Cauchy stress tensor are the principal stresses, i.e. $\sigma = \sigma(\sigma_r, \sigma_\theta, \sigma_z)$ where σ_r denotes the principal radial stress, σ_θ the principal circumferential stress and σ_z the principal axial stress.

1.1 Deformation

In the light of the above assumptions, the deformation gradient tensor becomes

$$\mathbf{F}^w = \mathrm{diag}\left[\frac{\partial r}{\partial R}, \frac{r}{R}, \frac{l_a}{L_a}\right], \tag{S1}$$

where R and r are the radial coordinates of an arbitrary point within the arterial wall in the reference and deformed state, respectively. L_a and l_a are the undeformed and deformed arterial length, respectively.

The principal stretches describing the deformation are expressed as

$$\lambda_r = \frac{\partial r}{\partial R}, \quad \lambda_\theta = \frac{r}{R}, \quad \lambda_\zeta = \frac{l_a}{L_a}. \tag{S2}$$

Here, λ_r, λ_θ, and λ_ζ denote the principal stretches in the radial, circumferential and axial direction, respectively, recalling that λ_ζ is a known constant from the experimental study of Bell et al. (2013). Due to the incompressibility property of the arterial wall, the principal stretches must satisfy the condition

$$\lambda_r \lambda_\theta \lambda_\zeta = 1, \tag{S3}$$

which yields a relationship between the undeformed and deformed radius of the artery, as follows. Substituting the expressions S2 into the condition S3, gives the differential equation

$$\frac{dr}{dR} = \frac{R}{\lambda_\zeta r} \tag{S4}$$

and after integrating the above

$$\int_{r_i}^{r} r dr = \frac{1}{\lambda_\zeta} \int_{R_i}^{R} R dR, \tag{S5}$$

a relationship between the undeformed radius R and the deformed radius r is obtained

$$r = \sqrt{r_i^2 + \frac{R^2 - R_i^2}{\lambda_\zeta}}, \tag{S6}$$

where r varies between the deformed inner radius r_i and the deformed outer radius r_o. The only input of the model is the reference configuration (e.g. R_i and R_o), the constant arterial pressure P and the constant axial stretch λ_ζ. The deformed state of the system is completely unknown. Therefore, in order to calculate the stresses and strains in the artery wall under physiological conditions, the deformed inner radius r_i must be determined first. The derivation of the equation that requires solving in order to determine r_i is given below.

1.2 Force-balance equation

The balance of the generated forces during the deformation of the artery wall is captured in the conservation of momentum equation, given here at a spatial point in the Eulerian framework. In the absence of time-dependence and any body forces, the conservation of momentum equation reduces to the

force-balance equation

$$\nabla \cdot \sigma = 0, \tag{S7}$$

where ∇ denotes the spatial divergence of the Cauchy stress σ in the deformed configuration of the system. Considering the assumed form of the Cauchy stress, e.g. $\sigma = \sigma(\sigma_r, \sigma_\theta, \sigma_z)$, the only non-trivial component of Equation S7, in cylindrical coordinates, is

$$\frac{d\sigma_r}{dr} + \frac{\sigma_r - \sigma_\theta}{r} = 0, \tag{S8}$$

with boundary conditions

$$\sigma_r|_{r=r_i} = -P \quad \text{and} \quad \sigma_r|_{r=r_o} = 0, \tag{S9}$$

that impose continuity of stress at the inner and outer boundaries of the artery wall. A constant arterial pressure P is applied on the inner boundary of the wall, while zero pressure is assumed on the outer boundary.

A commonly employed approach for modelling active arteries (Kalita and Schaefer, 2008; Rachev and Hayashi, 1999) is to calculate the total circumferential stress as the sum of passive stress and active stress

$$\sigma_\theta = \sigma_\theta^p + \sigma_\theta^a, \tag{S10}$$

where σ_θ^p denotes the passive stress and σ_θ^a denotes the active stress.

1.2.1 Passive stresses

The passive Cauchy stresses are determined from a Fung-type strain energy function (reviewed by Kalita and Schaefer (2008)), which for the particular assumptions made here (e.g. incompressible, no torsion, radial inflation and longitudinal extension) reduces to

$$W = \frac{1}{2}c(e^Q - 1) \quad \text{with} \quad Q = c_1E_\theta^2 + c_2E_z^2 + 2c_3E_\theta E_z, \tag{S11}$$

where W denotes the strain energy function, i.e. the stored elastic energy. E_θ and E_z refer to the principal Green strains in the circumferential and axial directions, defined as $E_\theta = \frac{1}{2}(\lambda_\theta - 1)^2$ and $E_z = \frac{1}{2}(\lambda_\zeta - 1)^2$, respectively. The material parameters c [kPa], c_1, c_2 and c_3 were fit to the inflation and extension measurement data for passive middle cerebral arteries (Bell et al., 2013) and are given in Table S1. Despite its phenomenological nature, the Fung-type strain energy function compares well with some microstructure-based constitutive models of arteries, such as the Holzapfel model and its extension to the 4-fibre family model (Hu et al., 2007). Expression S11 reveals that only two of the three principal components of stress are independent and determined from the strain energy function Humphrey (2013). The third component (e.g. σ_r) can also be determined by imposing equilibrium and boundary conditions, as it will be shown below. Hence, the chosen strain energy function still describes the three-dimensional nature of the system and allows us to impose incompressibility directly by giving the differences between the three principal

stresses as

$$\sigma_\theta^p - \sigma_r = \lambda_\theta^2 \frac{\partial W}{\partial E_\theta}, \tag{S12}$$

$$\sigma_z - \sigma_r = \lambda_\zeta^2 \frac{\partial W}{\partial E_z}. \tag{S13}$$

1.2.2 The active stress

The choice of a model for the active cerebral artery is difficult because the previous active models contain a high number of fitted parameters and none of them have been validated for cerebral arteries. For simplicity reasons, we assume that the VSMCs generate an active force only in the circumferential direction and choose the active model from Rachev and Hayashi (1999) according to which the active circumferential stress is

$$\sigma_\theta^a = S\lambda_\theta f(\lambda_\theta) \quad \text{with} \quad f(\lambda_\theta) = 1 - \left(\frac{\lambda_m - \lambda_\theta}{\lambda_m - \lambda_0}\right)^2. \tag{S14}$$

Here, S reflects the level of VSMC contractility and its values were determined by Rachev and Hayashi (1999) based on experimental recordings of deformations of rat carotid arteries with relaxed and contracting VSMCs (Cox and Bockus Institute, 1978). The value S = 0 kPa corresponds to the passive state (fully relaxed VSMCs), $S = 50$ kPa to the basal muscular tone and $S = 100$ kPa to the maximal muscular contraction (Rachev and Hayashi, 1999). By considering these values in the present arterial wall model, we assume that the VSMCs of the rat middle cerebral artery develop the same contractile force as those in the rat carotid artery. λ_m is the circumferential stretch at which maximum active contraction may develop and λ_0 is the minimum stretch possible at which active force can be generated. Here, we take $\lambda_m = 1.5$ and $\lambda_0 = 0.8$ as in Humphrey and Na (2002). The model from Equation S14 shows that the VSMCs are able to generate an active force only at certain lengths. For example, if the VSMC is over-stretched or over-contracted, it will not be able to generate any active stress. This property is captured in the length-tension relationship $f(\lambda_\theta)$ which describes a parabolic curve and whose qualitative behaviour is in line with more recent studies that accounted for the intracellular mechanisms leading to VSMC contraction (Murtada et al., 2012).

1.2.3 The pressure equation

The first step towards calculating the deformation at the middle of the artery wall and the corresponding radial stress (i.e. r_m and Σ) is to solve the arterial wall model for the deformed inner radius r_i. This is achieved by employing the local radial equilibrium from Equation S8 with the boundary conditions S9. Both radial and circumferential stresses are known functions of the deformed radius r, i.e. $\sigma_r = \sigma_r(r)$ and $\sigma_\theta = \sigma_\theta(r)$, where r varies between the inner radius r_i and outer radius r_o. It is recalled that also $r_o = r_o(r_i)$ according to Equation S6. Rearranging and integrating the equilibrium equation from Equation S8 over the thickness of the wall, gives

$$\sigma_r|_{r=r_o} - \sigma_r|_{r=r_i} = \int_{r_i}^{r_o} (\sigma_\theta^a + \sigma_\theta^p - \sigma_r)\frac{dr}{r}, \tag{S15}$$

with the boundary conditions

$$\sigma_r|_{r=r_i} = -P \quad \text{and} \quad \sigma_r|_{r=r_o} = 0. \tag{S16}$$

After substituting the boundary conditions S16 in the left-hand side of Equation S15 and replacing the expressions for the active stress from Eq. S14 and the passive stresses from Equation S12 in the right hand side of Equation S15, the pressure equation is obtained

$$P = \int_{r_i}^{r_o} \left(\lambda_\theta^2 \frac{\partial W}{\partial E_\theta} + S\lambda_\theta f(\lambda_\theta) \right) \frac{dr}{r}, \tag{S17}$$

where

$$r_o(r_i) = \sqrt{r_i^2 + \frac{R_o^2 - R_i^2}{\lambda_\zeta}}, \quad E_\theta = \frac{1}{2}\left(\lambda_\theta^2 - 1\right), \quad \lambda_\theta = \frac{r}{R}.$$

The pressure equation S17 plays a critical role in describing the behaviour of the system and is solved for r_i. Essentially, every time the deformation of the artery is assessed, one will return to Equation S17 to calculate the deformed inner radius r_i and, only subsequently, the strains and stresses within the wall will be determined.

1.2.4 Stresses within the arterial wall.

Once r_i is determined from Equation S17, the principal radial stress at any radial position r within the artery wall is calculated by integrating Equation S8 between r_i and any r and applying the first boundary condition in S9; this gives

$$\sigma_r(r) = \int_{r_i}^{r} \left(\lambda_\theta^2 \frac{\partial W}{\partial E_\theta} + S\lambda_\theta f(\lambda_\theta) \right) \frac{dr}{r} - P. \tag{S18}$$

By considering Equations S10, S12, S14 and S18, the expression for the total circumferential stress is then obtained as

$$\sigma_\theta(r) = \sigma_r + \lambda_\theta^2 \frac{\partial W}{\partial E_\theta} + S\lambda_\theta f(\lambda_\theta). \tag{S19}$$

The radial and circumferential stresses within the artery wall are shown in Supplementary Figure 1

1.3 Summary of the arterial wall model to calculate $r_m(z,t)$ and $\Sigma(z,t)$.

The radial position of the BM in the middle of the artery wall is defined as $r_m = (r_i + r_o)/2$. The corresponding radial Cauchy stress at the middle of the artery wall represents the constrictive stress Σ acting upon the BM, i.e. $\sigma_r|_{r=r_m} = \Sigma$. The dependence of r_m and Σ on the z (axial) coordinate and time t is only from the level of vascular tone $S(z,t)$, i.e. $r_m(z,t) = r_m(S(z,t))$ and $\Sigma(z,t) = \Sigma(S(z,t))$. The form we choose for $S(z,t)$ is discussed in detail in the main text (see Equation 7).

For a given value of S, the recipe to calculate $r_m(S)$ and $\Sigma(S)$ is summarized below

$$r_m(S) = \frac{r_i(S) + r_o(r_i(S))}{2}, \tag{S20}$$

where the deformed inner radius r_i is calculated from the pressure equation

$$P = \int_{r_i(S)}^{r_o(r_i(S))} \left(\lambda_\theta^2 \frac{\partial W(E_\theta, E_z)}{\partial E_\theta} + S\lambda_\theta f(\lambda_\theta) \right) \frac{dr}{r}, \tag{S21}$$

with

$$W(E_\theta, E_z) = \frac{1}{2} c(e^{c_1 E_\theta^2 + c_2 E_z^2 + 2c_3 E_\theta E_z} - 1), \quad E_\theta = \frac{1}{2}\left(\lambda_\theta^2 - 1\right), \quad E_z = \frac{1}{2}\left(\lambda_\zeta^2 - 1\right), \tag{S22a}$$

$$r_o(r_i(S)) = \sqrt{r_i(S)^2 + \frac{R_o^2 - R_i^2}{\lambda_\zeta}}, \quad f(\lambda_\theta) = 1 - \left(\frac{\lambda_m - \lambda_\theta}{\lambda_m - \lambda_0}\right)^2, \quad \lambda_\theta = \frac{r}{R}. \tag{S22b}$$

The terms R ($R_i \leq R \leq R_o$), P, λ_ζ, λ_m, λ_0, c, c_1, c_2 and c_3 are the input parameters (with no (z,t)-dependence) of the arterial wall model.

The radial stress at the middle of the artery wall is

$$\Sigma(S) = \int_{r_i(S)}^{r_m(S)} \left(\lambda_\theta^2 \frac{\partial W(E_\theta, E_z)}{\partial E_\theta} + S\lambda_\theta f(\lambda_\theta) \right) \frac{dr}{r} - P. \tag{S23}$$

The dependence of r_m and Σ on the level of vascular tone S, at a fixed point within the artery wall, is shown in Supplementary Figure 2; $r_m(S)$ and $\Sigma(S)$ represent the input of the BM model from the main text.

2 NUMERICAL SOLUTION OF THE BM MODEL: METHOD OF LINES

It is recalled from the main text that the equation of the BM model to be solved numerically is

$$\frac{\partial h}{\partial t} = \frac{\partial}{\partial z} \left(h \frac{k(h)}{\eta} \frac{\partial p(h, \Sigma(z,t))}{\partial z} \right), \tag{S24}$$

with

$$k(h) = k_* \left(\frac{\frac{h}{H} - \phi_*^s}{1 - \phi_*^s} \right)^\kappa \quad \text{and} \quad p = \sigma_y^e(z,t;h) - \Sigma(z,t;S),$$

where H and h denote the undeformed and deformed thickness of the upper-half BM, respectively. k is the BM permeability, η the fluid viscosity, p the fluid pressure, σ_e^y the effective Cauchy stress and Σ the external stress. k_* is the permeability of the undeformed BM and ϕ_*^s is the solid volume fraction in the fluid-filled undeformed state.

The initial condition is

$$h|_{t=0} = H, \quad 0 \leq z \leq L_s \tag{S25}$$

and the boundary conditions are

$$p|_{z=0} = p|_{z=L_s} = 0, \quad t > 0, \tag{S26}$$

where L_s denotes the length of the BM system.

Before applying the method of lines to this problem, Equation S24 is rewritten in terms of z, t and $h(z,t)$ only and a simpler notation is adopted, as follows. Firstly, we recall from the main text the strain energy function W_{BM}

$$W_{BM}\left(\frac{h}{H}\right) = \frac{\mu_s}{2}\left(\left(\frac{h}{H}\right)^2 - 1 - 2(1-\phi_*^s)\log\frac{\frac{h}{H}-\phi_*^s}{1-\phi_*^s}\right) + \frac{1}{2}\left(\lambda_s - \mu_s\frac{\phi_*^s}{1-\phi_*^s}\right)\left(\frac{h}{H}-1\right)^2, \quad \text{(S27)}$$

where the material parameters μ_s and λ_s denote the first and second Lam$\acute{e}$ parameters, respectively. Then we determine the expression of the effective Cauchy stress σ_e^y by deriving W_{BM} with respect to the ratio h/H. Further, the resulting σ_e^y is derived with respect to z giving

$$\frac{\partial \sigma_y^e}{\partial z}\left(\frac{h}{H}\right) = \left(\mu_s\frac{1-\phi_*^s}{(\frac{h}{H}-\phi_*^s)^2} + \mu_s\frac{1-2\phi_*^s}{1-\phi_*^s} + \lambda_s\right)\frac{\partial\left(\frac{h}{H}\right)}{\partial z}. \quad \text{(S28)}$$

Substituting Equation S28 in Equation S24 and denoting $\hat{h} = \frac{h}{H}$, the parabolic equation for $h(z,t)$ becomes

$$\frac{\partial \hat{h}}{\partial t} = \frac{k_*}{\eta}\left(\frac{\partial}{\partial z}\left(\mathcal{A}\frac{\partial \hat{h}}{\partial z}\right) - \mathcal{C}\frac{\partial \mathcal{B}}{\partial z} - \mathcal{B}\mathcal{D}\right), \quad \text{(S29)}$$

where, for visual purposes, the following notation was made

$$\mathcal{A}(\hat{h}(z,t)) = \hat{h}\left(\frac{\hat{h}-\phi_*^s}{1-\phi_*^s}\right)^{\kappa}\left(\mu_s\frac{1-\phi_*^s}{(\hat{h}-\phi_*^s)^2} + \mu_s\frac{1-2\phi_*^s}{1-\phi_*^s} + \lambda_s\right), \quad \text{(S30a)}$$

$$\mathcal{B}(\hat{h}(z,t)) = \hat{h}\left(\frac{\hat{h}-\phi_*^s}{1-\phi_*^s}\right)^{\kappa}, \quad \text{(S30b)}$$

$$\mathcal{C}(z,t) = \frac{\partial \Sigma}{\partial z}, \quad \text{(S30c)}$$

$$\mathcal{D}(z,t) = \frac{\partial^2 \Sigma}{\partial z^2}. \quad \text{(S30d)}$$

The initial condition S25 is rewritten in terms of $\hat{h}$ as

$$\hat{h}|_{t=0} = 1, \quad 0 \le z \le L_s \quad \text{(S31)}$$

and, by accounting for Equation S28, the boundary conditions S26 become

$$0 = \mu_s\left(\hat{h} - \frac{1-\phi_*^s}{\hat{h}-\phi_*^s}\right) + \left(\lambda_s - \mu_s\frac{\phi_*^s}{1-\phi_*^s}\right)(\hat{h}-1) - \Sigma(0,t), \quad \text{for} \quad t>0 \quad \text{and} \quad z=0, \quad \text{(S32)}$$

$$0 = \mu_s\left(\hat{h} - \frac{1-\phi_*^s}{\hat{h}-\phi_*^s}\right) + \left(\lambda_s - \mu_s\frac{\phi_*^s}{1-\phi_*^s}\right)(\hat{h}-1) - \Sigma(1,t), \quad \text{for} \quad t>0 \quad \text{and} \quad z=L_s. \quad \text{(S33)}$$

By employing the method of lines, the parabolic partial differential equation S29 is approximated by a set of coupled ordinary differential equations (ODEs) owing to the fact that the spatial derivatives are discretised using second order accurate finite differences on an uniform computational grid. In this instance, a uniform computational grid with $N+1$ grid points is used, such that the total domain $z \in [0, L_s/L]$ is partitioned into N subintervals, where L_s denotes the length of the system and L the characteristic length (taken to be the wavelength of vasomotion). The grid points are given by z_i for $0 \leq i \leq N$ and the approximated BM thickness at each point is denoted by $h_i(t) \approx h(z_i, t)$.

Using central differences, the first derivative is approximated as

$$h_z = \frac{1}{\Delta z}\delta_z h_i(t) = \frac{h_{i+1/2} - h_{i-1/2}}{\Delta z} \tag{S34}$$

and the second derivative as

$$h_{zz} = \frac{1}{\Delta z^2}\delta_z(\delta_z h_i(t)) = \frac{h_{i+1} - 2h_i + h_{i-1}}{\Delta z^2}, \tag{S35}$$

where δ_z is the central difference operator and Δz is the spacing between points. The values of h at the intermediate grid points can be approximated using the known values at the adjacent grid points:

$$h_{i+1/2} \approx \frac{h_{i+1} + h_i}{2} \quad \text{and} \quad h_{i-1/2} \approx \frac{h_{i-1} + h_i}{2}, \tag{S36}$$

where $i = 1, ..., N-1$.

Using expressions S34-S36, Equation S29 is discretised as

$$\begin{aligned}
\frac{d\hat{h}_i(t)}{dt} &= \frac{k_*}{\eta}\left(\frac{1}{\Delta z^2}\delta_z(\mathcal{A}_i\delta_z\hat{h}_i) - \frac{\mathcal{C}_i}{\Delta z}\delta_z\mathcal{B}_i - \mathcal{B}_i\mathcal{D}_i\right) \\
&= \frac{k_*}{\eta}\left(\frac{1}{\Delta z^2}\delta_z(\mathcal{A}_i(\hat{h}_{i+1/2} - \hat{h}_{i-1/2}))\right) - \frac{k_*}{\eta}\left(\frac{\mathcal{C}_i}{\Delta z}(\mathcal{B}_{i+1/2} - \mathcal{B}_{i-1/2}) - \mathcal{B}_i\mathcal{D}_i\right) \\
&\approx \frac{k_*}{\eta}\left(\frac{1}{\Delta z^2}(\mathcal{A}_{i+1/2}(\hat{h}_{i+1} - \hat{h}_i) - \mathcal{A}_{i-1/2}(\hat{h}_i - \hat{h}_{i-1}))\right) - \frac{k_*}{\eta}\left(\frac{\mathcal{C}_i}{2\Delta z}(\mathcal{B}_{i+1} - \mathcal{B}_{i-1}) - \mathcal{B}_i\mathcal{D}_i\right) \\
&\approx \frac{k_*}{\eta}\frac{1}{2\Delta z^2}\left(\mathcal{A}_{i+1}(\hat{h}_{i+1} - \hat{h}_i) + \mathcal{A}_i(\hat{h}_{i+1} - 2\hat{h}_i + \hat{h}_{i-1}) + \mathcal{A}_{i-1}(\hat{h}_{i-1} - \hat{h}_i)\right) \\
&\quad - \frac{k_*}{\eta}\left(\frac{\mathcal{C}_i}{2\Delta z}(\mathcal{B}_{i+1} + \mathcal{B}_{i-1}) + \mathcal{B}_i\mathcal{D}_i\right) \\
&\text{for} \quad i = 1, ..., N-1 \quad \text{and} \quad t > 0.
\end{aligned} \tag{S37}$$

The following notation was adopted: $\hat{h}_i = \hat{h}_i(t) = \hat{h}(z_i, t)$, $\mathcal{A}_i = \mathcal{A}(\hat{h}_i)$, $\mathcal{B}_i = \mathcal{B}(\hat{h}_i)$, $\mathcal{C}_i = \mathcal{C}(z_i, t)$ and $\mathcal{D}_i = \mathcal{D}(z_i, t)$. The values of $\hat{h}(z_i, t) = \hat{h}_i$ for $i = 1, ..., N-1$ are obtained by solving the system of ODEs from S37. The values of $\hat{h}$ at the ends of the computational domain are denoted $\hat{h}(z_0, t) = \hat{h}_0$ and $\hat{h}(z_N, t) = \hat{h}_N$ and can be determined explicitly (i.e. unnecessary to calculate updated values) from the boundary conditions S32 and S33, respectively, which become

$$\mu_s\left(\hat{h}_0 - \frac{1 - \phi_*^s}{\hat{h}_0 - \phi_*^s}\right) + \left(\lambda_s - \mu_s\frac{\phi_*^s}{1 - \phi_*^s}\right)(\hat{h}_0 - 1) = \Sigma(z_0, t), \quad \text{for} \quad t > 0, \tag{S38}$$

$$\mu_s \left(\hat{h}_N - \frac{1 - \phi_*^s}{\hat{h}_N - \phi_*^s} \right) + \left(\lambda_s - \mu_s \frac{\phi_*^s}{1 - \phi_*^s} \right) (\hat{h}_N - 1) = \Sigma(z_N, t), \quad \text{for} \quad t > 0, \tag{S39}$$

where $\Sigma(z_0, t)$ and $\Sigma(z_N, t)$ are known functions.

The temporal integration of the ODE system S37 is solved in MATLAB using the solver *ode15s*; this has an adaptive time step and so overcomes stability issues associated with discretizing Equation S24. The spatial grid on which computations are performed is long enough in order to allow for evaluation of the solution far away from the extremes of the spatial grid where rapid variations in the solution may occur. The convergence of the employed numerical method is tested by refining the spatial grid, repeating all the computations and checking the changes in results; here, case 1 from Table S1 was checked. In particular, the pointwise convergence of the scheme is demonstrated by interrogating the value of h at the end of one temporal cycle of vasomotion in two distinct positions, the beginning and middle of the wavelength, respectively, as well as the value of the fluid flow rate at the end of the wavelength. These values are chosen because, regardless of the number of grid points employed, they are easily accessible following temporal integration without requiring additional interpolation. Given the lack of exact solutions to the problem, the convergence is verified by calculating the error relative to a solution computed on a highly refined grid. Here, the maximum number of points considered is denoted N_M (e.g. $N_M = 4000$) and the corresponding solution, assumed to be the most accurate one, is denoted $g^{(N_M)}$ where g could be any of the three quantities aforementioned. The following error is defined

$$\epsilon(N) = |g^{(N)} - g^{(N_M)}| \tag{S40}$$

where the quantity g (e.g. h or Q_{BM}) is computed using N points with $N \ll N_M$. The pointwise convergence of g is shown in Supplementary Figure 3, demonstrating the second order convergence of the scheme.

3 THE HHCY ANIMAL MODEL

Hyperhomocystaeinaemia (HHcy) was induced in wild-type C57/Bl6 mice as in the study of Sudduth et al. (2014). Mice of 10 weeks old (n=5) were perfusion fixed with 4% paraformaldehyde and cryoprotected sequentially in 10, 20, and 30% sucrose (24 hours each). Brains were frozen, sectioned on a sliding microtome at 25μm thickness, collected floating in PBS+sodium azide and stored at 4° C. For double labelling immunohistochemistry, tissue sections were incubated overnight with anti-α-smooth muscle actin (SMA) FITC (1:200, Sigma-Aldrich, Dorset, UK) for the identification of arteries. Anti-collagen IV (1:400, ABCAM, Cambridge, UK) was used to mark the basement membranes of blood vessels (Thermo Fisher Scientific, Paisley, UK). Sections were incubated with the goat anti-rabbit conjugated to Alexafluor546 fluorophores. Sections were analysed using an SP8 confocal laser-scanning microscope (Milton Keys, UK) and exported to Image J software.

The expression of smooth muscle actin (SMA) immunostaining in the leptomeningeal vessels of both the HHcy group (n=5) and control group (n=7) was assessed. The percentage area occupied by SMA immunostaining was analyses using Student t-test. Despite the lack of statistical significance (p=0.072), there was a strong meaningful trend of lower expression of SMA in the HHcy models compared to the control group, as illustrated in Supplementary Figure 4.

REFERENCES

Bell, E. D., Kunjir, R. S., and Monson, K. L. (2013). Biaxial and failure properties of passive rat middle cerebral arteries. *Journal of biomechanics* 46, 91–96

Cox, R. H. and Bockus Institute, P., Philadelphia (1978). Comparison of carotid artery mechanics in the rat, rabbit, and dog. *American Journal of Physiology-Heart and Circulatory Physiology* 234, H280–H288

Hu, J.-J., Baek, S., and Humphrey, J. (2007). Stress–strain behavior of the passive basilar artery in normotension and hypertension. *Journal of biomechanics* 40, 2559–2563

Humphrey, J. and Na, S. (2002). Elastodynamics and arterial wall stress. *Annals of biomedical engineering* 30, 509–523

Humphrey, J. D. (2013). *Cardiovascular solid mechanics: cells, tissues, and organs* (Springer Science & Business Media)

Kalita, P. and Schaefer, R. (2008). Mechanical models of artery walls. *Archives of Computational Methods in Engineering* 15, 1–36

Murtada, S. C., Arner, A., and Holzapfel, G. A. (2012). Experiments and mechanochemical modeling of smooth muscle contraction: significance of filament overlap. *Journal of theoretical biology* 297, 176–186

Rachev, A. and Hayashi, K. (1999). Theoretical study of the effects of vascular smooth muscle contraction on strain and stress distributions in arteries. *Annals of biomedical engineering* 27, 459–468

Sudduth, T. L., Weekman, E. M., Brothers, H. M., Braun, K., and Wilcock, D. M. (2014). β-amyloid deposition is shifted to the vasculature and memory impairment is exacerbated when hyperhomocysteinemia is induced in app/ps1 transgenic mice. *Alzheimer's research & therapy* 6, 32

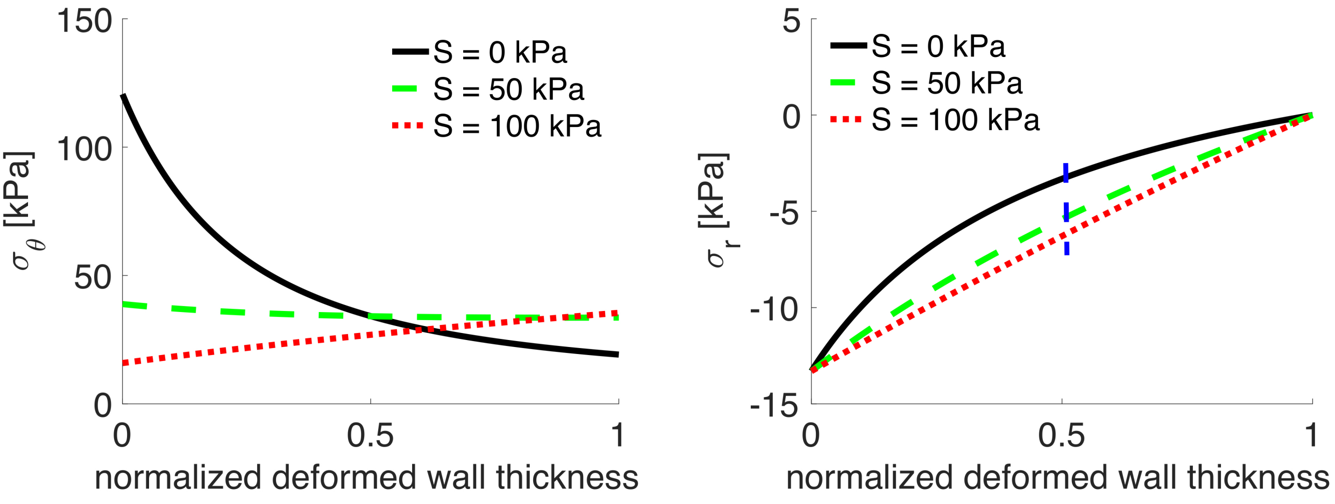

Supplementary Figure 1. Stress distribution within the artery wall for an artery exposed to an intramural pressure $P = 13.3$ kPa, an axial stretch $\lambda_\zeta = 1.07$ and different levels of vascular tone: the total circumferential stress σ_θ (left-hand side) and the radial stress σ_r (right-hand side). The distribution of the total circumferential stress becomes (nearly) uniform under conditions of basal and maximal vascular tone. The radial stress that represents the external constrictive stress acting upon the BM membrane is the one from the middle of the artery wall, as emphasized by the vertical dotted blue line.

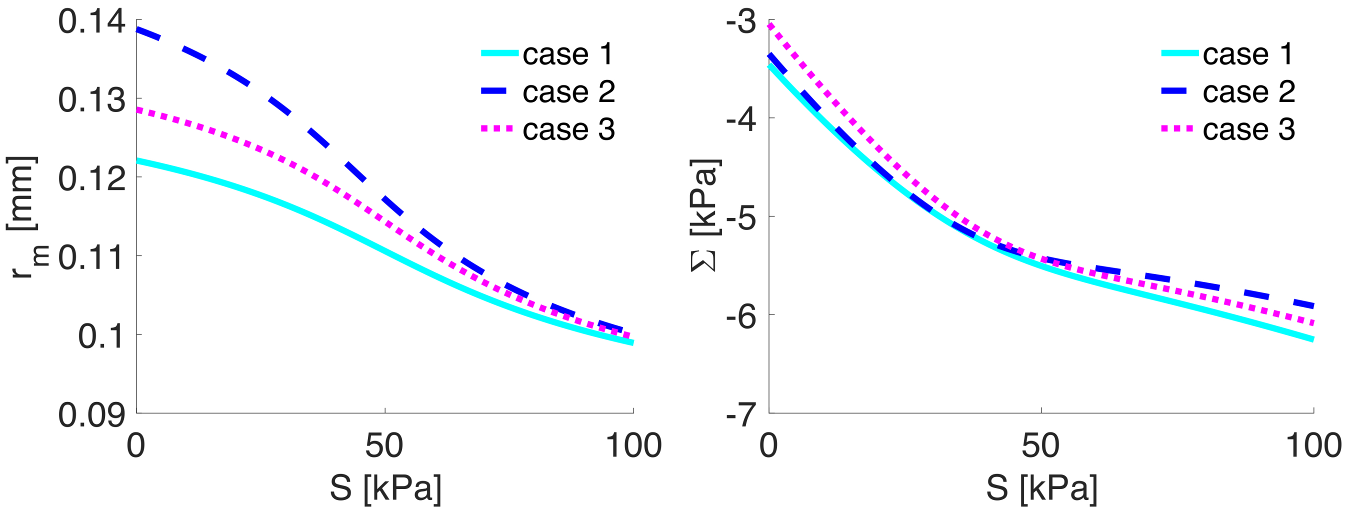

Supplementary Figure 2. The deformation (left-hand side) and corresponding radial stress (right-hand side) at the middle of the artery wall as a function of the vascular tone S. Three middle cerebral arteries are investigated, each of them experiencing a distinct axial stretch: $\lambda_\zeta = 1.07$ (case 1), $\lambda_\zeta = 1.13$ (case 2) and $\lambda_\zeta = 1.09$ (case 3).

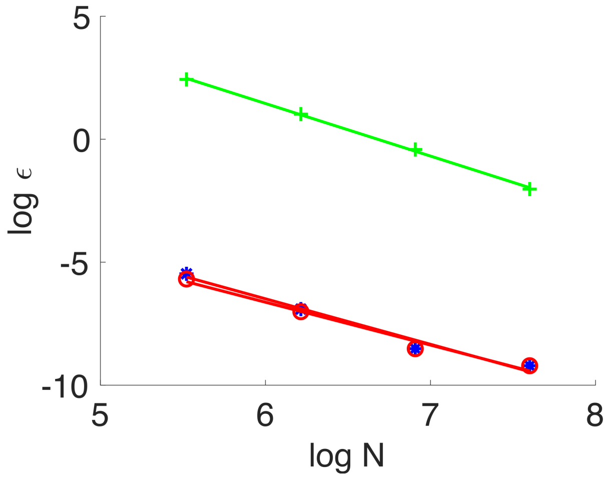

Supplementary Figure 3. Illustration of pointwise convergence of the numerical method. The pointwise error ϵ is shown against the number of grid points N for the deformed BM thickness h at the beginning (blue stars) and middle (red open circles) of a wavelength and for the fluid flow rate at the end of the wavelength (green cross). The markers give the errors for computations with $N = 250$, 500, 1000 and 2000, respectively, against the numerical solution with N_M = 4000 grid points. The lines show second order convergence of the method.

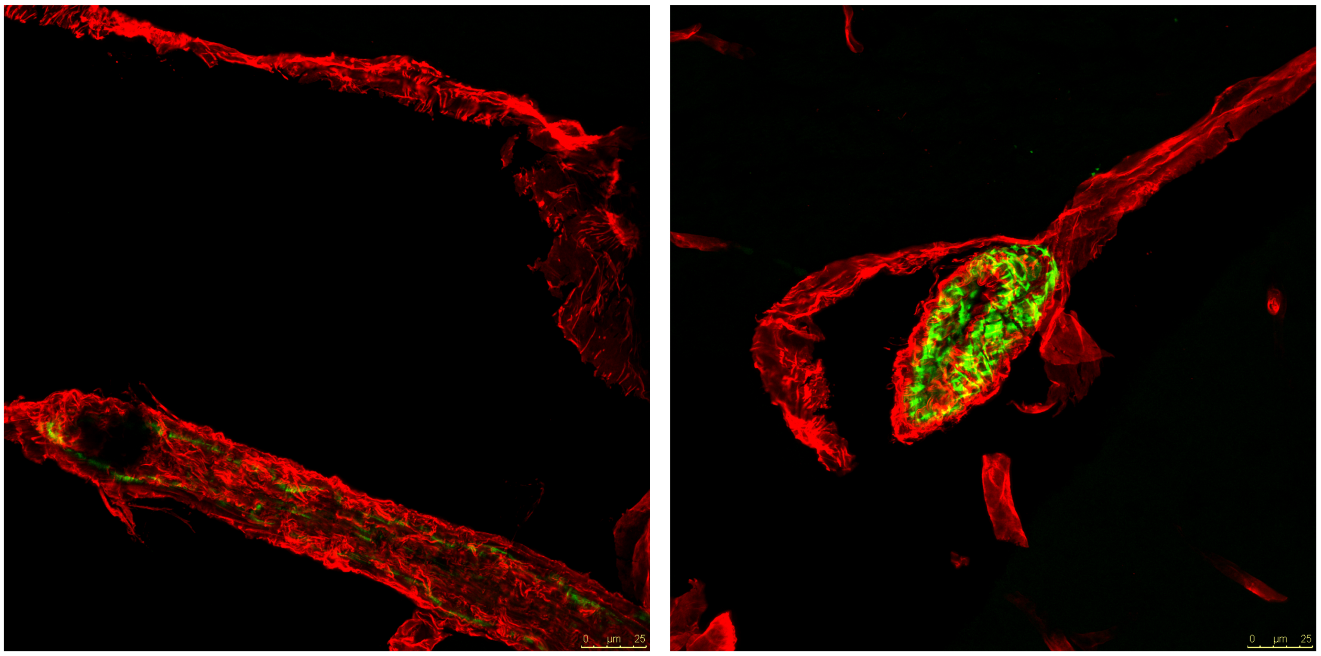

Supplementary Figure 4. Leptomeningeal vessels of HHcy (left-hand side) and control (right-hand side) mouse brain sections. Collagen IV (red) reveals the vascular basement membrane and smooth muscle actin (SMA, green) shows smooth muscle in the tunica media of the vessel wall. Control vessel shows bright staining for SMA while HHcy shows comparatively reduced SMA expression. Scale bars = 25 μm.

Table S1. Material parameters from Bell et al. (2013) for three passive arteries exposed to different levels of axial stretch.

Case	c [kPa]	c_1	c_2	c_3	λ_ζ
1	5.59	12.25	37.03	6.16	1.07
2	13.19	3.12	12.47	0.48	1.13
3	4.94	8.71	40.78	0.85	1.09
